# Additively manufactured unimorph dielectric elastomer actuators: Design, materials, and fabrication

**DOI:** 10.3389/frobt.2022.1034914

**Published:** 2022-12-16

**Authors:** Stanislav Sikulskyi, Zefu Ren, Danayit T. Mekonnen, Aleiya Holyoak, Rishikesh Srinivasaraghavan Govindarajan, Daewon Kim

**Affiliations:** ^1^ Department of Aerospace Engineering, Embry-Riddle Aeronautical University, Daytona Beach, FL, United States; ^2^ Department of Mechanical Engineering, Embry-Riddle Aeronautical University, Daytona Beach, FL, United States

**Keywords:** unimorph dielectric elastomer actuator (UDEA), bending actuator, modeling, figure of merit, material selection, 3D printing, viscosity, manufacturability

## Abstract

Dielectric elastomer actuator (DEA) is a smart material that holds promise for soft robotics due to the material’s intrinsic softness, high energy density, fast response, and reversible electromechanical characteristics. Like for most soft robotics materials, additive manufacturing (AM) can significantly benefit DEAs and is mainly applied to the unimorph DEA (UDEA) configuration. While major aspects of UDEA modeling are known, 3D printed UDEAs are subject to specific material and geometrical limitations due to the AM process and require a more thorough analysis of their design and performance. Furthermore, a figure of merit (FOM) is an analytical tool that is frequently used for planar DEA design optimization and material selection but is not yet derived for UDEA. Thus, the objective of the paper is modeling of 3D printed UDEAs, analyzing the effects of their design features on the actuation performance, and deriving FOMs for UDEAs. As a result, the derived analytical model demonstrates dependence of actuation performance on various design parameters typical for 3D printed DEAs, provides a new optimum thickness to Young’s modulus ratio of UDEA layers when designing a 3D printed DEA with fixed dielectric elastomer layer thickness, and serves as a base for UDEAs’ FOMs. The FOMs have various degrees of complexity depending on considered UDEA design features. The model was numerically verified and experimentally validated through the actuation of a 3D printed UDEA. The fabricated and tested UDEA design was optimized geometrically by controlling the thickness of each layer and from the material perspective by mixing commercially available silicones in non-standard ratios for the passive and dielectric layers. Finally, the prepared non-standard mix ratios of the silicones were characterized for their viscosity dynamics during curing at various conditions to investigate the silicones’ manufacturability through AM.

## 1 Introduction

**FIGURE 1:**
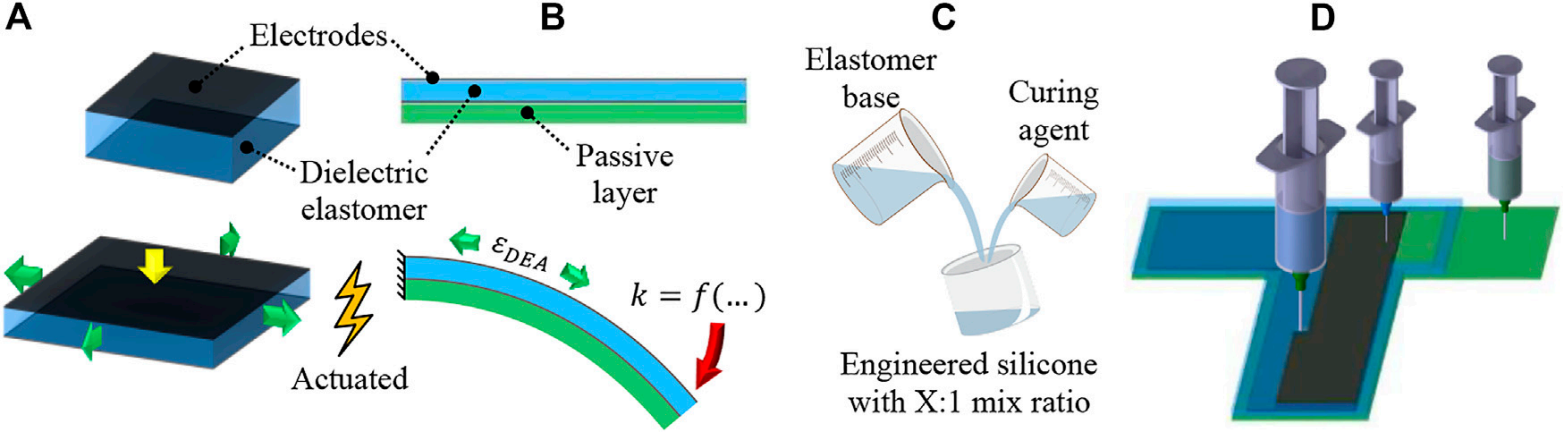
Structure and actuation mode of a **(A)** planar and **(B)** unimorph DEA configurations, **(C)** silicone modification through various mix ratios of elastomer base and curing agent, and **(D)** 3D printing of each UDEA layer through contact dispensing (direct ink writing).

Dielectric elastomer actuator (DEA) is one of the electroactive polymers that has attracted immense interest in the field of soft robotics and electronics due to its fast and reversible electromechanical response, large strain capabilities, and high specific energy density (Duduta et al., 2019; Hajiesmaili and Clarke, 2021). DEA consists of a dielectric elastomer (DE) sandwiched between two compliant electrodes and operates through an electrostatic pressure mechanism when high voltage is applied to the electrodes. For the planar DEA, actuation mode occurs in the form of thickness contraction that is effectively translated to in-plane expansion due to typical elastomer materials being nearly incompressible (Figure 1A). To enhance deflection capabilities, the unimorph DEA (UDEA) configuration possesses an additional external passive layer that translates DEA’s in-plane expansion into the bending of the entire actuator (Figure 1B). As a result, considerably larger deflection can be achieved by UDEA at lower strains (Kim et al., 2022).

The planar DEA actuation performance is driven by its materials and layers’ thicknesses and is often described with one of the figures of merit (FOM) as in Eq. 1, which shows the amount of actuation strain in the thickness direction (McKay et al., 2009).

$$FOM\;\left(\max\;strain\;for\;planar\;DEA\right)=\frac{\varepsilon_{o}\varepsilon_{r}E_{B}^{2}}{Y_{DE}}$$ (1)

where $\varepsilon_{o}$ is the permittivity constant, $\varepsilon_{r}$ is relative permittivity, $E_{B}$ is dielectric breakdown strength, and $Y_{DE}$ is Young’s modulus of DE material. As per the FOM, DE material’s dielectric and mechanical properties drive the maximum possible DEA actuation while DE thickness determines the voltage required to enable the actuation as applied electric field is the ratio of voltage over DE thickness, $E=V/t_{DE}$ . Despite neglecting electrode thickness and assuming DE material be linearly elastic, planar DEA FOMs allow for an uncomplicated estimation of actuator performance and material selection (Sikulskyi et al., 2022).

Meanwhile, modeling UDEA is more complicated than planar DEAs from the layers’ design standpoint and lacks a simple tool like FOM for material selection and design optimization despite numerous static and dynamic analytical modeling approaches with elastic, hyperelastic, and viscoelastic DE materials (Lau et al., 2011; Kadooka et al., 2016; Kadooka, 2017; Haghiashtiani et al., 2018; Gareis and Maas, 2021; Hajiesmaili and Clarke, 2021). Besides accounting for DE layer properties mentioned above, the thickness and modulus of each layer, including the passive layer and electrodes, contribute to overall multilayer beam stiffness. Additionally, soft unimorph actuators largely adopt their modeling approaches from piezoelectric unimorph actuators (Wang and Cross, 1998; Ballas, 2007; Araromi and Burgess, 2012). As a result, numerous effects, such as electrode stiffness, large deflection, and deformation under weight, are often overlooked in the unimorph actuator design process. Therefore, there is a need for a simple analytical model that considers important features of soft unimorph actuators with reasonable simplifications and UDEA FOM that allows simplified material selection and design optimization.

Table 1 shows 3D printed and conventionally fabricated UDEAs found in literature, their reported material characterization and actuation performance, and potential optimized design performance based on the model derived and validated in this paper. Only actuators with known parameters and conventional unimorph structure are reported in the table, despite the achieved large actuation of some other actuators (Lau et al., 2011; Pu et al., 2022). In the table, some deviation between experimental curvatures and analytical FOM can be noticed for some studies which is attributed to material characterization and thickness measurements of UDEA layers. The table shows that single- and multilayer UDEA in numerous studies achieved high values of analytical curvatures of the fabricated designs relative to the maximum possible curvatures of the optimized design. However, the passive layer thicknesses were far from optimum values for most of studies despite actuators’ high performance. That is explained by gently sloped FOM curves with respect to passive layer thickness, when passive layer has comparable Young’s modulus to the DE layer. Meanwhile, performance of actuators with thin and stiff passive layers is compromised considerably if far from optimum passive layer thicknesses are used (Araromi and Burgess, 2012; Sikulskyi et al., 2020).

**TABLE 1:** Tested unimorph DEA designs in literature and their optimized performance.

Year [Ref.]	Studied designs in literature					Optimized	
	DE layer(s)	Passive layer^a^	Electrodes	Curvature (m^−1^)⸸ x thickness (µm)	Analytical curvature (FOM)⸸⸸	Passive layer thickness (µm)	Curvature (m^−1^) (% of max FOM reached)⸸
3D printed							
2016 (Kadooka et al., 2016)	10 layers of 15 μm P(VDF-TrFE-CFE) polymer	63 µm 3M 810 Magic Tape (Y_p_ = 1.66 GPa)	12 µm MWCNT/PDMS composite (Y_ele_ = 4.9 MPa)	8 m^−1^ × 345 µm = 0.00276	4.17 m^−1^	46 µm	4.23 m^−1^ (98.6%)
	(Y_DE_ = 300 MPa, er = 50, V_max_ = 550V)						
2018 (Haghiashtiani et al., 2018)	516 µm Loctite 5039/Semicosil 912/BaTiO_3_ composite (Y_DE_ = 39.8 kPa, er = 4.16, V_max_ = 5.44kV)	313 µm (Y_p_ = 168 kPa)	304 and 458 µm hydrogel ink (Y_ele_ = 8.8 kPa)	41.7 m^−1^ × 1591 µm = 0.0663	39.7 m^−1^	179 µm	41.4 m^−1^ (95.9%)
2020 (Sikulskyi et al., 2020)	90 µm Sylgard 184 PDMS (Y_DE_ = 1 MPa, er = 2.63, V_max_ = 2.4kV)	25.4 µm Kapton film (Y_p_ = 2.1 GPa)	10 µm PEDOT:PSS/Triton X-100 (Y_ele_ = 6 MPa)	14.7 m^−1^ × 135.4 µm = 0.002	11.7 m^−1^	2.5 µm	39.4 m^−1^ (30%)
2022 (Sikulskyi et al., 2022)	300 µm Sylgard 184 (15:1 mix ratio) PDMS/CaCu_3_Ti_4_O_12_ composite	350 µm	20 µm PEDOT:PSS/Triton X-100 (Y_ele_ = 3.5 MPa)	9.73 m^−1^ × 680 µm = 0.0066	8.04 m^−1^	237 µm	8.58 m^−1^ (93.7%)
	(YDE = 0.761 MPa, er = 3.75, Vmax = 5.2 kV)						
This paper (Design 1)	446 µm Sylgard 182 PDMS	304 µm	20 µm PEDOT:PSS/Triton X-100 (Y_ele_ = 3.5 MPa)	9.37 m^−1^ × 780 µm = 0.00731	—	—	—
This paper (Design 2)	521 µm Sylgard 182 (30:1 mix ratio) PDMS	234 µm Sylgard 182 (10:1 mix ratio) PDMS	20 µm PEDOT:PSS/Triton X-100 (Y_ele_ = 3.5 MPa)	24.1 m^−1^ × 785 µm = 0.0189	—	—	—
“Conventionally” fabricated							
2012 (Araromi and Burgess, 2012)	4 layers of 89 µm Dow Corning 3481 with 81-F curing agent (Y_DE_ = 0.56 MPa, er = 3.68, V_max_ = 3.54 kV)	50 µm steel (Y_p_ = 200 GPa)	1 µm sprayed graphite (Y_ele_ = 10 MPa)^b^	3.6 m^−1^ × 410 µm = 0.00125	0.88 m^−1^	0.65 µm	83.9 m^−1^ (1.05%)
2016 (Duduta et al., 2016)	12 layers of 30 µm CN9021 with 10% HDDA crosslinker (Y_DE_ = 1.8 MPa, er = 3, V_max_ = 1.75 kV)	12.5 µm Mylar (Y_p_ = 1 GPa)	100 nm SWCNT (Y_ele_ = 100 MPa)^b^	50 m^−1^ × 373 µm = 0.01865	77.3 m^−1^	9.5 µm	77.5 m^−1^ (99.7%)

^a^ Material is not specified for the passive layer if it is the same as of DE layer.

^b^ Exact values were not reported and were adopted from studies on similar materials. Curvature due to actuation only is considered (neglecting bending under weight). As per the derived FOM (single-layer) or the full analytical model for multilayer UDEAs.

Fabrication is another crucial aspect of DEA development due to its immense impact on the actuator performance. Besides, their multilayer and multimaterial structure makes DEA fabrication an effort- and time-consuming process. As a result, additive manufacturing (AM) or 3D printing is one of the promising techniques for automating DEA fabrication and enabling complex biomimetic soft actuators (Chen et al., 2021; Kim et al., 2022). Therefore, characterizing material manufacturability is an important aspect of high-performance DEA design implementation through 3D printing. For instance, viscosity is one of the primary properties of uncured elastomer that enables printing, determines printing parameters, and drives the printed material rheology through traditional dispensing and newly developed techniques (Chortos et al., 2020; Schlatter et al., 2020; Chortos et al., 2021; Jiang et al., 2021; Regis et al., 2021; Shintake et al., 2021; Li et al., 2022; Naniz et al., 2022). In the literature, viscosity and storage shear modulus are measured after material preparation. However, there is a lack of studies on how material viscosity changes directly after mixing and during crosslinking (curing) process. Therefore, investigation of the post-mixing viscosity dynamics and manufacturability of common elastomers for 3D printing of soft robotics is an important supporting analysis of this study. To perform the analysis, several polydimethylsiloxanes (PDMS) elastomer compositions were mixed and characterized for their rheology and practical handling time for the contact dispensing AM method. PDMS Sylgard 184 was used as the material is commonly utilized in soft material technologies. Two similar PDMS materials, i.e. Sylgard 186 and Sylgard 182, were also studied due to its additional softness (186) and its long curing time (182), relatively underutilized characteristic allowing more prolonged 3D printing session without replacing the material. As a method to improve DEA electromechanical actuation, different mixing ratios of elastomer base (Part A) and platinum-based curing agent (Part B) were investigated for Sylgard 182 (Figure 1C) (Chen et al., 2019; Vaicekauskaite et al., 2019). Various optimized UDEA designs were 3D printed and tested to validate the derived model, FOM, and materials’ printability (Figure 1D).

## 2 Results and discussion

### 2.1 Analytical modeling

The derivation of an analytical model starts with analyzing the real actuator’s behavior and discussing what simplifications and assumptions are adequate to implement into the mathematical model. Major aspects to consider here are material, geometry, and loads.

*Material*. Due to the bending type of deformation, UDEAs produce large deflection at relatively low strain. Particularly, some of the best performing UDEAs produce considerable deformations with the bending angle up to 270° at the tip of the actuator while the maximum strain does not reach 5% in the actuator (Pu et al., 2022). While being material sensitive, such strains do not deviate the compressive modulus from its initial zero-strain value. Therefore, a linear material model can be implemented for most materials besides those that can behave very nonlinearly at the abovementioned low strain values.

*Geometry*. Several important aspects need to be discussed and addressed with respect to the geometry of UDEAs:

• *Structure type*. Having layers that are much thinner than their width and length, UDEAs are essentially plate-like structures. Thus, in-plane actuation of the DE layer results in UDEA curvature in both directions. While curvature along the longer side of UDEAs creates the desired deflection of the actuator, the transverse curvature increases its stiffness and negatively affects actuation capabilities. Furthermore, the plate’s bending stiffness is larger than the beam stiffness (Ugural, 2017). UDEA can be modeled as a plate structure to account for these effects. However, most UDEAs have a moderately larger length than width to effectively produce curvature in the desired direction. Hence, modeling UDEA as multilayer beams is a case-sensitive application but is appropriate for most designs in the literature (Table 1). Therefore, this work models UDEAs as beams to result in an overcomplicated analytical model and FOM.
• *Beam model.* As mentioned above, UDEAs are typically thin beams with high length-to-thickness ratios. Thus, an Euler-Bernoulli beam model and its negligence of shear stress are appropriate to model UDEA.
• *Unimorph DEA current configuration*. During actuation, DEA reduces its thickness which is accounted in Maxwell pressure equation by utilizing a current thickness of DE film. When UDEA actuates, this thinning reduces actuator’s bending stiffness and is an important factor to account for UDEAs.
• *Geometrical nonlinearity*. Numerous UDEAs have demonstrated large deflection and curvature capabilities thanks to their bending nature, material softness, and thin beam geometry. Accounting for geometrical nonlinearities such as large rotation and deflection is not trivial in some cases. In this study, large deflection is not considered as it is not needed to derive the FOM for unimorph DEA but is essential for its accurate modeling.
• *Insulation material*. It is almost always considered that electrodes occupy the entire area of DE films. However, practically all DEAs require insulation material to prevent the formation of a through-the-air circuit between the electrodes due to the high voltage applied. Despite the required amount of the insulation material being small and close to the DE layer thickness, much larger insulation can be practically implemented depending on the fabrication technique.

*Loads*. Typical materials for DEA include silicones, acrylic elastomers, rubbers, and more rarely stiffer dielectric materials, as well as compliant electrodes. The passive layers of UDEAs can often be made of stiffer material but usually are made thin not to hinder actuation capabilities. As a result, UDEAs typically bend considerably under their weight, making it one of the preferred loads to be accounted in the modeling.

#### 2.1.1 Small deflection analytical model

**FIGURE 2:**
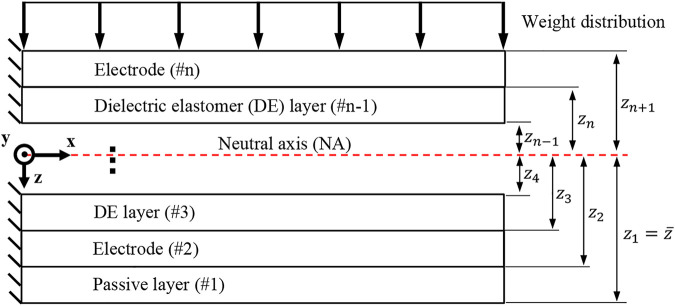
Multilayer unimorph DEA structure.

The derivation starts with the Euler-Bernoulli beam model, which assumes the planes perpendicular to the beam’s neutral axis (NA) to stay perpendicular upon bending. Correspondingly, longitudinal strain, ε , can be expressed through the thickness of the actuator, which also suggests deformation compatibility between the layers of UDEA (Eq. 2):

$$\varepsilon =-z\kappa^{\circ }$$ (2)

where z is distance from the bottom of the UDEA to the points where strain, ε , is calculated, $\kappa^{\circ }$ is the curvature of the actuator. According to the selected coordinates, positive curvature means positive deflection along z, i.e., down (Figure 2). Therefore, DEA actuation results in positive curvature and deflection.

Meanwhile, longitudinal strain in each layer of material of UDEA, i.e., passive layer, DE layers, and electrodes, can be shown as a summation of the mechanical elastic strain due to deformation compatibility between the layers, $\varepsilon_{mech,i}$ , and longitudinal strain due to DEA actuation, $\varepsilon_{DEA,i}$ , (Eq. 3). Note that the DEA strain component is present in DE layers only that are compressed by compliant electrodes.

$$\varepsilon_{i}=\varepsilon_{mech,i}+\varepsilon_{DEA,i}$$ (3)

The mechanical strain in the *i*th layer is simply the mechanical stress over the material’s Young’s modulus, $\varepsilon_{mech,i}=\sigma_{i}/Y_{i}$ , The DEA longitudinal strain can be calculated from the thickness strain through the Poisson’s deformation relation, as explained below. When voltage is applied to a DEA, its thickness is reduced, further increasing the DE layer’s electric field. To account for that, the thickness strain equation, $\varepsilon_{DEA,t,i}=-\frac{p}{Y}=-\frac{\varepsilon_{o}\varepsilon_{r}}{Y}{\left(\frac{V}{t_{i}}\right)}^{2}$ , is typically solved numerically in the current configuration, i.e., where $t_{i}$ is DE layer thickness in deformed state. To keep the analysis in the analytical form, an approximate analytical solution that has proved its accuracy can be utilized to calculate the longitudinal DEA strain as shown in Eq. 4 (Pelrine et al., 1998).

$$\varepsilon_{DEA,i}=v_{i}∙\varepsilon_{DEA,t,i}$$ (4)

where

$$\varepsilon_{DEA,t,i}=-\frac{2}{3}+\frac{1}{3}\left(f\left(\varepsilon_{DEA,\;t,oi}\right)+\frac{1}{f\left(\varepsilon_{DEA,t,oi}\right)}\right)$$

where

$$f\left(\varepsilon_{DEA,t,oi}\right)={\left[\frac{1}{2}\left(2+27\varepsilon_{DEA,t,oi}+\sqrt{-4+{\left(2+27\varepsilon_{DEA,t,oi}\right)}^{2}}\right)\right]}^{1/3}$$

where $\varepsilon_{DEA,t,oi}=\frac{p_{i}}{Y_{i}}=\frac{\varepsilon_{o}\varepsilon_{ri}}{Y_{i}}{\left(\frac{V}{t_{oi}}\right)}^{2}$ where $v_{i}$ is material’s Poisson’s ratio, $p_{i}$ is electrostatic (Maxwell) pressure, $Y_{i}$ is Young’s modulus, $\varepsilon_{ri}$ is the relative dielectric permittivity, and $t_{oi}$ is the initial (reference) thickness of *i*th layer, and V is the applied voltage. Note that Eq. 4 is Poisson’s strain relation but does not have the minus sign because DEA thickness strain is calculated as a positive value despite representing contracting deformation. Therefore, the thickness of each layer in the following equations is then calculated as $t_{i}=t_{oi}∙\left(1-\varepsilon_{DEA,t,i}\right)$ .

Once the relation between the strains in each layer is established as $-z\kappa^{\circ }=\varepsilon_{mech,i}+\varepsilon_{DEA,i}$ , force and moment equilibrium need to be satisfied for the multilayer beam considering its weight and DEA actuation (Eqs 5, 6, respectively):

$$\{\sum F=\overset{n}{\underset{i=1}{\sum }}b_{i}\overset{z_{i+1}}{\underset{z_{i}}{\int }}\sigma_{i}dz=\overset{n}{\underset{i=1}{\sum }}b_{i}Y_{i}\overset{z_{i+1}}{\underset{z_{i}}{\int }}\left(-z\kappa^{\circ }-\varepsilon_{DEA,i}\right)dz=0$$ (5)

$$\sum M=\overset{n}{\underset{i=1}{\sum }}b_{i}\overset{z_{i+1}}{\underset{z_{i}}{\int }}\sigma_{i}zdz-\frac{\omega }{2}{\left(L-x\right)}^{2}=\overset{n}{\underset{i=1}{\sum }}b_{i}\overset{z_{i+1}}{\underset{z_{i}}{\int }}Y_{i}\left(-z\kappa^{\circ }-\varepsilon_{DEA,i}\right)zdz-\frac{\omega }{2}{\left(L-x\right)}^{2}=0$$ (6)

where $z_{i}=\bar{z}-\overset{i-1}{\underset{j=1}{\sum }}t_{j}$ and $z_{i+1}=\bar{z}-\overset{i}{\underset{j=1}{\sum }}t_{j}$ are distances from the multilayer beam NA to the bottom and top surfaces of each layer, respectively.

Solving Eq. 5 for the NA location results in Eq. 7, which can be rewritten for curvature as in Eq. 8. Note that the NA location depends on both multilayer beam properties and DEA layer actuation. Similarly, Eq. 6 can be solved for curvature as in Eq. 9. The solution process is demonstrated in the Supplementary Material. Now, NA location, $\bar{z}$ , and curvature, $\kappa^{\circ }$ , can be found by either solving Eqs 7, 9, or Eqs 8, 9. Effects of weight and actuation on UDEA deflection and its NA locations under various load conditions are demonstrated in Supplementary Figure S1.

$$\bar{z}=\frac{\overset{n}{\underset{i=1}{\sum }}b_{i}Y_{i}t_{i}\left(\overset{i-1}{\underset{j=1}{\sum }}t_{j}+\frac{t_{i}}{2}-\frac{\varepsilon_{DEA,i}}{\kappa^{\circ }}\right)}{\overset{n}{\underset{i=1}{\sum }}b_{i}Y_{i}t_{i}}$$ (7)

$$\left\{ \begin{array}{c} \kappa^{\circ }=\frac{\overset{n}{\underset{i=1}{\sum }}b_{i}Y_{i}t_{i}\varepsilon_{DEA,i}}{\overset{n}{\underset{i=1}{\sum }}b_{i}Y_{i}t_{i}\left(\overset{i-1}{\underset{j=1}{\sum }}t_{j}+\frac{t_{i}}{2}-\bar{z}\right)} \end{array}\right.$$ (8)

$$\kappa^{\circ }=\frac{\frac{\omega }{2}{\left(L-x\right)}^{2}+\overset{n}{\underset{i=1}{\sum }}b_{i}Y_{i}t_{i}\varepsilon_{DEA,i}\left(\overset{i-1}{\underset{j=1}{\sum }}t_{j}+\frac{t_{i}}{2}-\bar{z}\right)}{\overset{n}{\underset{i=1}{\sum }}b_{i}Y_{i}t_{i}\left({\left(\overset{i-1}{\underset{j=1}{\sum }}t_{j}\right)}^{2}+t_{i}\overset{i-1}{\underset{j=1}{\sum }}t_{j}+\frac{t_{i}^{2}}{3}+{\bar{z}}^{2}-\bar{z}\left(2\overset{i-1}{\underset{j=1}{\sum }}t_{j}+t_{i}\right)\right)}$$ (9)

For the small deflection bending, the strain-deformation relation is expressed by Eqs 10, 11. The actuator’s deflection, w , and elongation, u , can be found through integrating each equation independently and applying cantilever boundary conditions.

$$\kappa^{\circ }=-d^{2}w/dx^{2}=>w\left(x\right)=-\overset{x}{\iint_{0}}\kappa^{\circ }dx^{2}$$ (10)

$$\varepsilon {}^{\circ}=\frac{du}{dx}$$ (11)

where ε° is elongation strain of the multilayer UDEA (average strain value across the thickness) due to the in-plane expansion of DE layers (Eq. 12). It can be found by dividing the force generated by DEA in the longitudinal direction by the total axial stiffness of the unimorph actuator. The force can be found as DE layer Young’s modulus times DE layer cross-section area times the longitudinal DEA actuation strain. Because the DEA actuation strain is zero in the layers other than DE, the “DE” indices can be dropped, as shown in Eq. 12. Finally, the elongation of the unimorph actuator along its length can be found, as shown in Eq. 13.

$$\varepsilon {}^{\circ}=\frac{F_{DEA}}{\sum A_{i}Y_{i}}=\frac{\sum_{i=1}^{n}Y_{DE,i}\varepsilon_{DEA,i}A_{DE,i}}{\sum_{i=1}^{n}A_{i}Y_{i}}=\frac{\sum_{i=1}^{n}Y_{i}A_{i}\varepsilon_{DEA,i}}{\sum_{i=1}^{n}A_{i}Y_{i}}$$ (12)

$$u\left(x\right)=\overset{x}{\underset{0}{\int }}\varepsilon {}^{\circ}dx=\frac{\sum_{i=1}^{n}Y_{i}A_{i}\varepsilon_{DEA,i}}{\sum_{i=1}^{n}A_{i}Y_{i}}∙x$$ (13)

**FIGURE 3:**
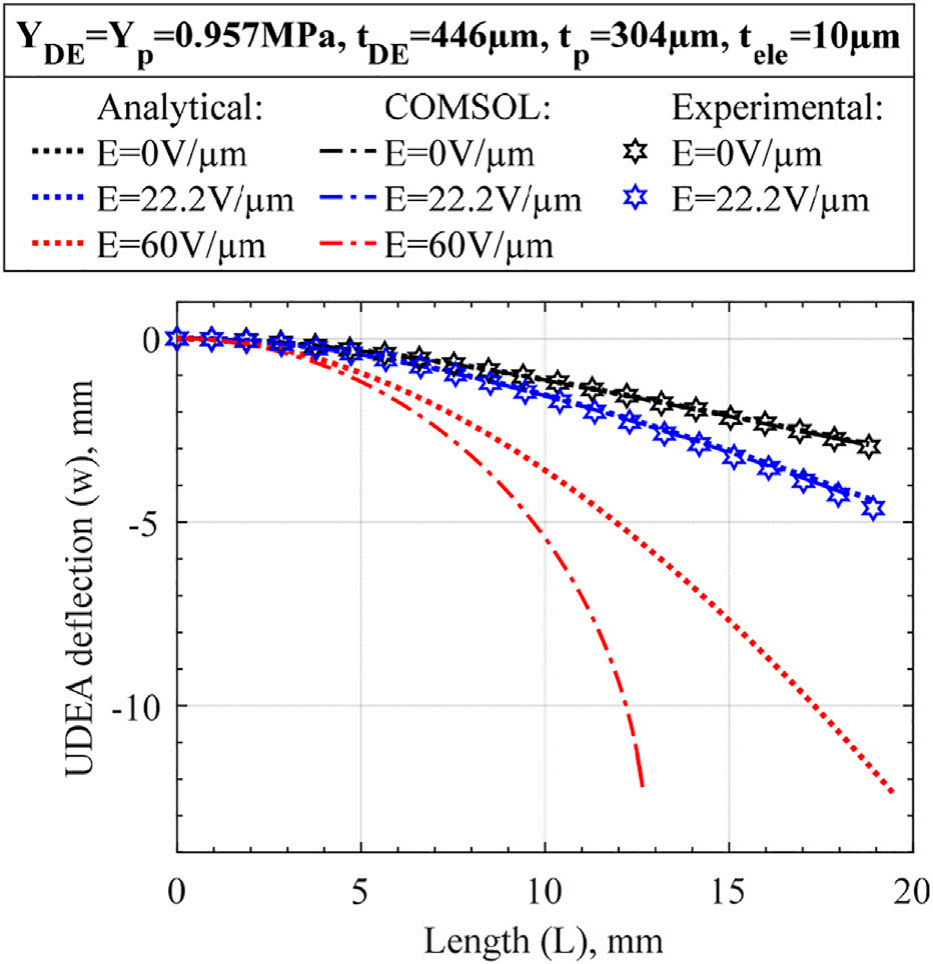
Deformed Unimorph DEA shape obtained using the derived small deflection analytical model and large deflection COMSOL Multiphysics FEM simulation for various loading/actuation cases. More actuation cases are presented in Supplementary Figure S2 to better illustrate the effect of large deflection on the selected UDEA design.

The derived analytical model was validated experimentally by comparing deflections of a selected UDEA design under the weight only and at maximum applied electric field of 22.2 V/μm reached in the experiment (Figure 3). The UDEA design produces moderate bending under its weight and has considerable actuation; its selection is shown in the result section. Both passive and DE layers were assumed to be made of Sylgard 182 (10:1) with PEDOT:PSS-based electrodes used in this study. The difference between analytical and experimental deflections are about 1% for the bending at no electric field, and less than 3% at the 22.2 V/μm. From the comparison without applied electric field, the practically identical deformed UDEA shapes suggest that the stiffness and weight loading of the multilayer beam are described correctly in the derived model. The larger difference for the actuated case was attributed to a larger deflection and corresponding geometrical nonlinearity, not considered in the analytical model. Therefore, a numerical modeling with a linear material models and accounting for geometrical nonlinearities was performed. Commercial finite element method (FEM) software COMSOL Multiphysics was utilized to obtain numerical solutions of the UDEA actuation in the cantilever mode. In the simulation, the passive layer and electrodes were modeled as linearly elastic materials, while the DE layer was modeled as a linear dielectric to enable electromechanical coupling. Same Young’s moduli of the materials were selected in the numerical simulation as in the analytical model as specified in the figures with Poisson’s ratio of 0.49. The comparison demonstrated a practically negligible difference between the numerical and analytical deflections at no electric field for the selected UDEA design. As the electric field of 22.2 V/μm was applied, the difference in deflections was still less than 1%. To illustrate the effect of large deflection geometrical nonlinearity, the electric field of 60 V/μm, the highest electric field reached for 3D printed DEAs in the literature, was applied in the numerical and analytical models (Schlatter et al., 2020). At 60 V/μm applied electric field, UDEA rotation becomes more apparent, violating linear beam geometry assumptions and considerably changing weight distribution along the actuator. Therefore, while for the selected UDEA design, the deflection at practically achievable electric field can be estimated through the derived analytical model with a small error, further UDEA actuation would result in a deformed shape that is predicted inaccurately and require geometrical nonlinearities consideration.

#### 2.1.2 Figure of merit

Following the derived analytical models, a FOM was derived to provide a straightforward tool to optimize the unimorph actuator design and quantify the performance of the passive, electrode, and DE layers’ materials. It is convenient to set the FOM to represent the curvature of the actuator that can be maximized without the concern of large deflection. Therefore, Eqs 8, 9 are used as the starting point to derive the uncomplicated FOM for UDEAs. Along the derivation, the following features and assumptions accounting for a typical UDEA structure are used to obtain and simplify the FOM expressions (the full derivation and additional comments are available in Supplemental Material):

1. Actuator’s weight can be neglected because the objective of FOM is to achieve maximum actuation.
2. Cross-section is rectangular with width, b , same across the passive, electrode, and DE layers, i.e., neglecting the isolation material:
3. A typical unimorph material layout starts with the passive layer and then alternates between the electrode and DE layers ( $n_{p}=1,\;n_{ele}=n_{DE}+1$ ).
4. All DE layers have the same thickness, and all electrodes have the same thickness.
5. Same voltage potential difference (voltage) is applied to all electrodes.
6. Actuation strain, $\varepsilon_{DEA,i}$ , happens in DE layers only.

According to the above assumptions, Eqs 8, 9 transform to Eqs 14, 15, respectively, for a multilayer unimorph actuator. However, an explicit expression for FOM cannot be obtained yet.

$$\kappa^{\circ }=\frac{n_{DE}Y_{DE}t_{DE}\varepsilon_{DEA}}{Y_{p}t_{p}\left(\frac{t_{p}}{2}-\bar{z}\right)+Y_{DE}t_{DE}n_{DE}\left(t_{p}+\frac{n_{DE}t_{DE}}{2}+\frac{\left(n_{DE}+1\right)t_{ele}}{2}-\bar{z}\right)+Y_{ele}t_{ele}\left(n_{DE}+1\right)\left(t_{p}+\frac{n_{DE}t_{DE}}{2}+\frac{\left(n_{DE}+1\right)t_{ele}}{2}-\bar{z}\right)}$$ (14)

$$\kappa^{\circ }=\frac{n_{DE}Y_{DE}t_{DE}\varepsilon_{DEA}\left(t_{p}+\frac{n_{DE}t_{DE}}{2}+\frac{\left(n_{DE}+1\right)t_{ele}}{2}-\bar{z}\right)}{Y_{p}t_{p}\left(\frac{t_{p}^{2}}{3}+{\bar{z}}^{2}-\bar{z}t_{p}\right)+Y_{DE}t_{DE}n_{DE}A+Y_{ele}t_{ele}\left(n_{DE}+1\right)B}$$ (15)

where

$$A=t_{p}^{2}+\frac{1}{3}n_{DE}^{2}t_{DE}^{2}+\frac{1}{6}\left(2n_{DE}^{2}+3n_{DE}+1\right)t_{ele}^{2}+n_{DE}t_{DE}t_{p}+\left(n_{DE}+1\right)t_{p}t_{ele}+\frac{1}{6}\left(4n_{DE}^{2}+3n_{DE}-1\right)t_{DE}t_{ele}+{\bar{z}}^{2}-2\bar{z}\left(t_{p}+\frac{n_{DE}t_{DE}}{2}+\frac{\left(n_{DE}+1\right)t_{ele}}{2}\right)$$

$$B=t_{p}^{2}+\frac{1}{6}\left(2n_{DE}^{2}+n_{DE}\right){\left(t_{DE}+t_{ele}\right)}^{2}+\left(\frac{n_{DE}}{2}+\frac{1}{3}\right)t_{ele}^{2}+n_{DE}t_{DE}t_{p}+\frac{1}{2}n_{DE}t_{DE}t_{ele}+\left(n_{DE}+1\right)t_{ele}t_{p}+{\bar{z}}^{2}-\bar{z}\left(2t_{p}+t_{ele}+n_{DE}\left(t_{DE}+t_{ele}\right)\right)$$

7. Furthermore, for a UDEA with a single DE layer ( $n_{DE}=1$ ), FOM can be explicitly expressed as the curvature as in Eq. 16:

$$\begin{array}{l} FOM\;\left(single-layer\right)=k\\ =\frac{6\mu \varepsilon_{r}\varepsilon_{o}E_{B}^{2}Y\left(t+t^{2}+2tR_{t}\right)}{Y_{DE}t_{DE}\left(1+Y^{2}t^{4}+R_{Y}R_{t}\left(8+12R_{t}+8R_{t}^{2}\right)+Yt\left(4+12R_{t}+6t+12R_{t}^{2}+12tR_{t}+4t^{2}\right)+R_{Y}^{2}R_{t}^{2}\left(12+24R_{t}+16R_{t}^{2}\right)+R_{Y}R_{t}Yt\left(12+36R_{t}+12t+32R_{t}^{2}+24tR_{t}+8t^{2}\right)\right)}\\ where=Y_{p}/Y_{DE},\;t=t_{p}/t_{DE},\;R_{Y}=Y_{ele}/Y_{DE},\;and\;R_{t}=t_{ele}/t_{DE} \end{array}$$ (16)

8. Finally, similarly to a commonly used FOM for planar DEAs, electrodes’ contribution can be neglected ( $Y_{ele}=0$ and $t_{ele}=0$ ) to obtain the equivalently simplified FOM for UDEAs as in Eq. 17.

$$FOM\;\left(no\;electrodes\right)=k=\frac{6\mu \varepsilon_{r}\varepsilon_{o}E_{B}^{2}Y\left(t+t^{2}\right)}{Y_{DE}t_{DE}\left(1+Y^{2}t^{4}+Yt\left(4+6t+4t^{2}\right)\right)}$$ (17)

Resulting in the optimum thickness-modulus ratio:


$$\frac{d\left(FOM\right)}{dY}=0\to t_{optimum}=\frac{1}{\sqrt{Y}}for\;the\;fixed\;total\;actuator\;thickness$$ (18)

$$\frac{d\left(FOM\right)}{dt}=0\to Y_{optimum}=\frac{1}{t^{2}\left(2t+3\right)}for\;the\;fixed\;DE\;thickness$$ (19)

Derived FOMs (Eqs 16, 17) represent the achievable curvature of the UDEA at the highest possible applied electric field, which is the breakdown strength of the DE material, $E_{B}$ . The breakdown in DE materials is a statistical event and, thus, does not guarantee reaching that value of electric field in every DEA (Carpi et al., 2015). Furthermore, 3D printed DEAs and UDEAs suffer from relatively low reachable electric fields (Kim et al., 2022). Therefore, it is worth keeping in mind that FOM serves as a comparative tool for various UDEA materials and designs while providing theoretical maximum actuator performance in terms of curvature.

Once the FOMs are derived for different degrees of UDEA simplification, the analysis of various design parameters on actuator’s performance is conducted in the following order. First, Eq. 17 shows the effect of DE and passive layers on UDEA performance and finds the optimum design while neglecting the electrode contribution. Then, Eq. 16 is used to show the stiffening effect of electrodes. Lastly, Eqs 14, 15 are used to show the effect of number of DE layers. Similarly to the material in the numerical analysis, the presented analysis of the derived FOM is performed with the DE material that has dielectric permittivity, $\varepsilon_{r}$ , of 2.6, breakdown strength, $E_{B}$ , of 100 V/μm, and the rest of parameters specified in the performed analysis below. FOM values, calculated at applied electric field equal to the DE breakdown strength, represent the maximum achievable curvature in m^−1^ by a UDEA design.

It is worth mentioning that the derived FOM and analytical model can be easily applied to unimorph actuator made of any material by replacing the DEA actuation strain $\varepsilon_{DEA}=\mu \varepsilon_{r}\varepsilon_{o}E_{B}^{2}/Y_{DE}$ , and DE parameters with the values of a different active material.

#### 2.1.3 Effects of DE and passive layers

**FIGURE 4:**
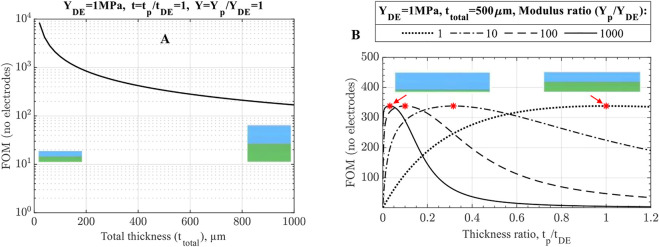
No-electrode FOM performance of unimorph DEA **(A)** for variable and **(B)** fixed total thickness. The optimum designs at $t=\sqrt{1/Y}$ are marked in **(B)** having the same maximum actuation curvature as determined by no-electrode FOM.

**FIGURE 5:**
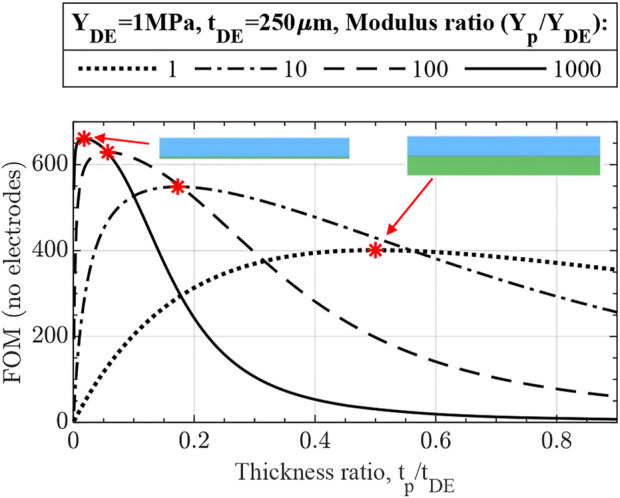
No-electrode FOM for unimorph DEA designs with fixed DE thickness demonstrating better performance of actuators with thinner and stiffer passive layers due to the smaller total thickness. The optimum designs are marked and match with the derived optimum ratio $Y=\frac{1}{t^{2}\left(2t+3\right)}$ .

Despite being vastly simplified, Eq. 17 demonstrates numerous essential elements of UDEA actuation performance:

• As Eq. 17 shows, curvature is a hyperbolic function of actuator thickness if electrodes are neglected. Therefore, total actuator thickness largely drives unimorph actuation performance in terms of its curvature (Figure 4A). Thus, if actuators with different total thicknesses are compared and judged in terms of optimized material properties, appropriate criteria is curvature multiplied by thickness as was stated in (Pu et al., 2022).
• For the fixed actuator’s total thickness, the optimum ratios of modulus and thickness are known from the previous studies on piezoelectric unimorph actuators as $t=\sqrt{1/Y}$ , which is in agreement with the derived expression in this work (Eq. 18) (Wang and Cross, 1998). Interestingly, if FOM is plotted for various combinations of modulus ratios, Y , and thickness ratios, t , but with the fixed total thickness, the optimum actuator designs result in the same FOM performance, i.e., curvature (Figure 4B). Nevertheless, the actuator design with a stiff and thin passive layer and thicker DE layer benefit from a larger blocked force and is therefore preferred.
• Furthermore, for DEAs, especially 3D printed ones, manufacturing is the primary limitation for obtaining a thin, high-quality DE film for low-voltage actuation. This is attributed to the fact that breakdown failure happens at the location of DE film with the smallest thickness or the presence of some defect, such as air bubbles, voids, or trapped dust. Therefore, a more practical approach is where the elastomer thickness is fixed at the minimum adequately printable value, and the optimum modulus-thickness ratio is determined through Eq. 19. Figure 5 shows that using a thinner and stiffer passive layer for the fixed DE layer increases unimorph actuator performance. Notably, the optimum design is now located at $Y=\frac{1}{t^{2}\left(2t+3\right)}$ , which is in a close agreement with the optimum design determined through a hyperelastic modeling of UDEA (Hajiesmaili and Clarke, 2021).
• Lastly, modifying DE material is another way of enhancing the actuation. From Eq. 17, it is apparent that increasing DE material’s relative dielectric permittivity or breakdown strength proportionally increases actuation performance. However, some methods to increase DE dielectric properties lead to material mechanical stiffening, e.g., through dielectric composites (Haghiashtiani et al., 2018; Pu et al., 2022; Sikulskyi et al., 2022). Per Eq. 17, the trade between DE modulus and dielectric properties can be expressed for a single-layer UDEA with completely compliant and thin electrodes as $\varepsilon_{r}E_{B}^{2}/Y_{DE}$ , which is identical to max actuation strain FOM for planar DEAs without the vacuum permittivity constant (Eq. 1). However, a UDEA with a DE layer possessing higher modulus and equivalent $\varepsilon_{r}E_{B}^{2}/Y_{DE}$ value (same appearance of Figure 5 with higher $Y_{DE}$ ) can have additional benefit for unimorph actuators like smaller bending under its weight. Moreover, while stiff passive layers (e.g., Y=1000 ) in Figure 5 require their thickness to be very thin and likely produced through advanced manufacturing techniques, commercially available films with moderately thin thicknesses can be used for actuators with composite DE layers. An alternative way to enhance unimorph actuator performance in terms of its curvature is to reduce the moduli of DE and passive layers proportionally. This approach is demonstrated experimentally and analytically further in this paper.

#### 2.1.4 Effects of electrodes

**FIGURE 6:**
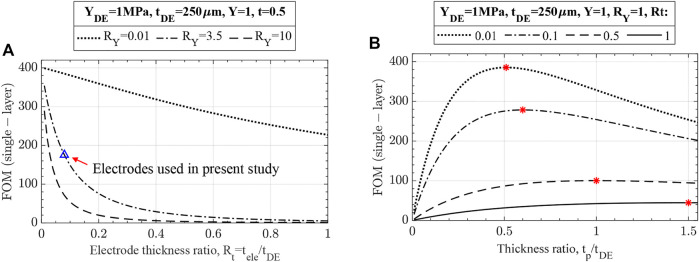
Effects of electrodes on unimorph DEA actuation evaluated through the single-layer FOM for unimorph DEAs with fixed **(A)** DE and passive layers, **(B)** modulus of all layers and DE thickness. For the fixed electrode, thickness and modulus are taken as for PEDOT:PSS-based electrodes used in present study.

The effects of electrodes on UDEA’s actuation and optimized designs are demonstrated through the single-layer UDEA FOM (Eq. 16). Figure 6A demonstrates performance of a unimorph actuator with a fixed design of DE, passive layers, and various electrode designs. The curve representing $R_{Y}=0.01$ suggests that thick electrodes considerably degrade actuator performance even when their modulus is negligible. As electrodes’ modulus increases, actuation performance degrades at higher rates for thinner electrodes. Meanwhile, performance impact of the electrodes used in the present study on the studied UDEA design is marked in the figure and corresponds to about 19% of actuation degradation compared to no-electrode actuator. Furthermore, Figure 6B demonstrates effect of electrodes on optimizing the UDEA geometry. For the analysis, moduli of all layers are fixed, replicating selected materials for passive, DE, and electrode layers. Meanwhile, passive layer and electrode thicknesses are altered relative to the fixed DE thickness. According to the determined optimized designs, optimized designs have considerably smaller actuation and require thicker passive layers when using thicker electrodes. Overall, the figures suggest that while electrodes need to be compliant, their thickness has a greater negative effect than its modulus, as could also be drawn from the FOM in Eq. 17. Similar analysis for UDEAs with fixed total thickness is demonstrated in.

#### 2.1.5 Effects of multilayer unimorph DEA

**FIGURE 7:**
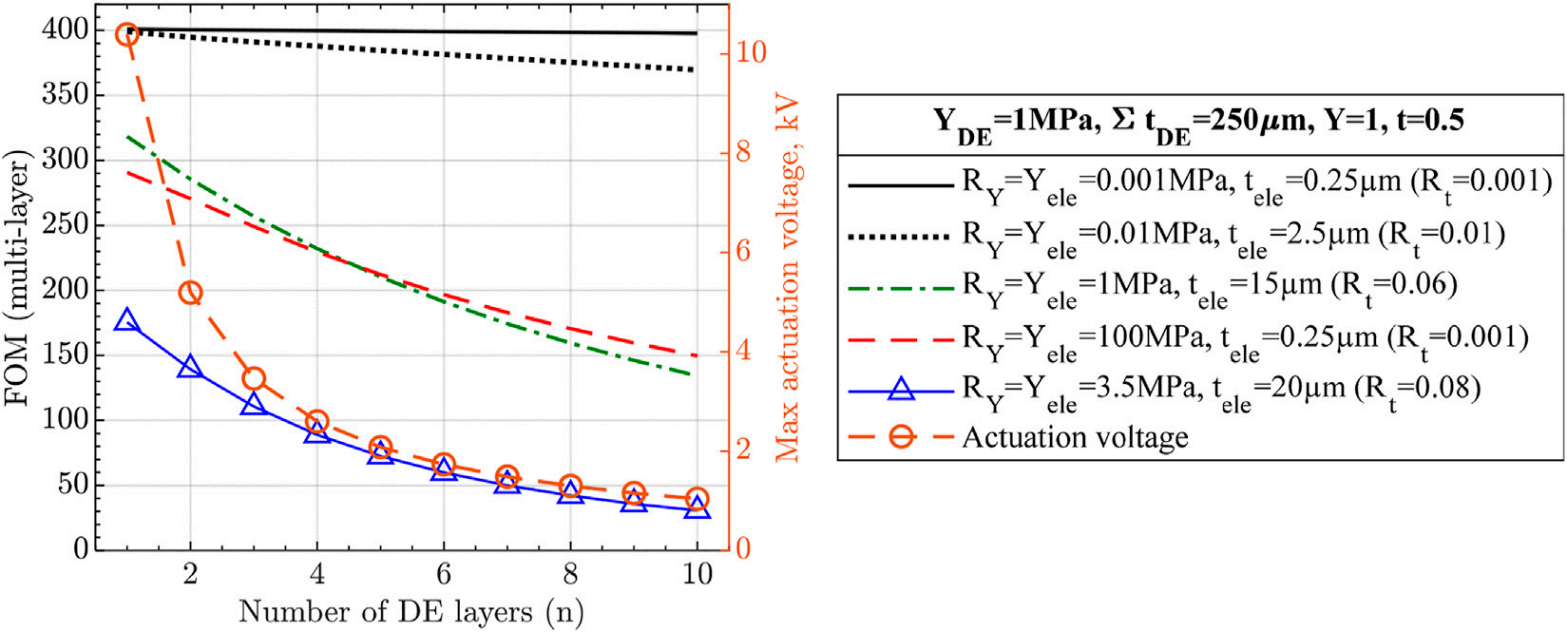
Actuation performance of multilayer unimorph DEA with various number of layers and electrodes. Cumulative thickness of all DE layers, passive layer thickness, and modulus for both DE and passive layers are fixed to demonstrate the reduced actuation voltage and the stiffening effect of electrodes for multilayer unimorph DEAs.

Now, the effects of number of DE layers on multilayer UDEA (MUDEA) are analyzed. The main benefit of breaking the DE layer into multiple layers with the same cumulative thickness is actuation at a reduced voltage that is inversely proportional to the thickness of the individual DE layer (Figure 7). However, each DE layer added to a single-layer UDEA requires adding another electrode layer, increasing overall actuator stiffness and reducing actuation performance. Based on Eqs 14, 15 and Figure 7 shows how electrodes degrade MUDEA actuation as number of DE layers increases:

• As the figure shows (and also shown in Figure 6A), electrodes with $R_{Y}=R_{t}=0.01$ has a negligible stiffening effect on single-layer UDEA. However, it reduces the actuation of the selected MUDEA design with 10 layers by approximately 7.5%. To prove the performance reduction comes solely from the electrodes’ stiffening, electrodes with $R_{Y}=R_{t}=0.001$ are evaluated demonstrating practically no change in actuation performance.
• The following two curves represent compliant-moderately thin electrodes ( $R_{Y}=1,\;R_{t}=0.06$ ) and stiff-thin electrodes ( $R_{Y}=100,\;R_{t}=0.001$ ). The results in the figure demonstrate that compliant-moderately thin electrodes might serve better for UDEAs with a smaller number of DE layers, ultra-thin electrodes provide better performance for the actuators with many layers despite their high stiffness.
• Lastly, the solid blue line with triangle markers demonstrates the performance of the actuator with electrodes utilized in this study.

### 2.2 Elastomer material selection

Selecting a dielectric material with an appropriate combination of electromechanical, handling, and manufacturability properties is crucial for actuators’ fabrication, performance, and application. Electromechanical properties for which the mixed silicone compositions were characterized include dielectric permittivity, breakdown strength, and Young’s modulus, which are later used in material selection through the derived FOMs and for modeling UDEA.

#### 2.2.1 Elastomer manufacturability and handling

First, material properties related to actuators’ fabrication and application are investigated for the mixed silicone compositions. As discussed in Introduction, viscosity is a parameter of uncured polymers that dominantly drives their printability through different techniques. For two-component RT cured polymers, such as Sylgard 182, 184, and 186, viscosity gradually increases once an elastomer base is mixed with its curing agent. For fabrication and printing process, it means that there is a limitation in terms of time within which material can be used. Often, manufacturers provide a materials’ handling time within which viscosity does not interfere with manufacturing processes, e.g., the time within which viscosity doubles. However, the definition of handling time can be different and is too generalized for various manufacturing processes. During different printing processes, material curing can be affected differently and locally, e.g., by the presence of a heating bed, material experiencing various shear strain rates, etc. Therefore, viscosity of the silicones studied in this work is measured through two tests. The first test is a standard procedure to measure viscosity of liquids and is used to study the changes in viscosity during curing, which can be used to analyze materials’ manufacturability for different fabrication techniques. Considering the above together with handling and curing times of the silicones, the test aimed to investigate material viscous behavior for up to 12 h after the mixing process to simulate daytime usage of the material. The second test is performed to study the viscosity change effect during curing on contact dispensing 3D printing process.

**FIGURE 8:**
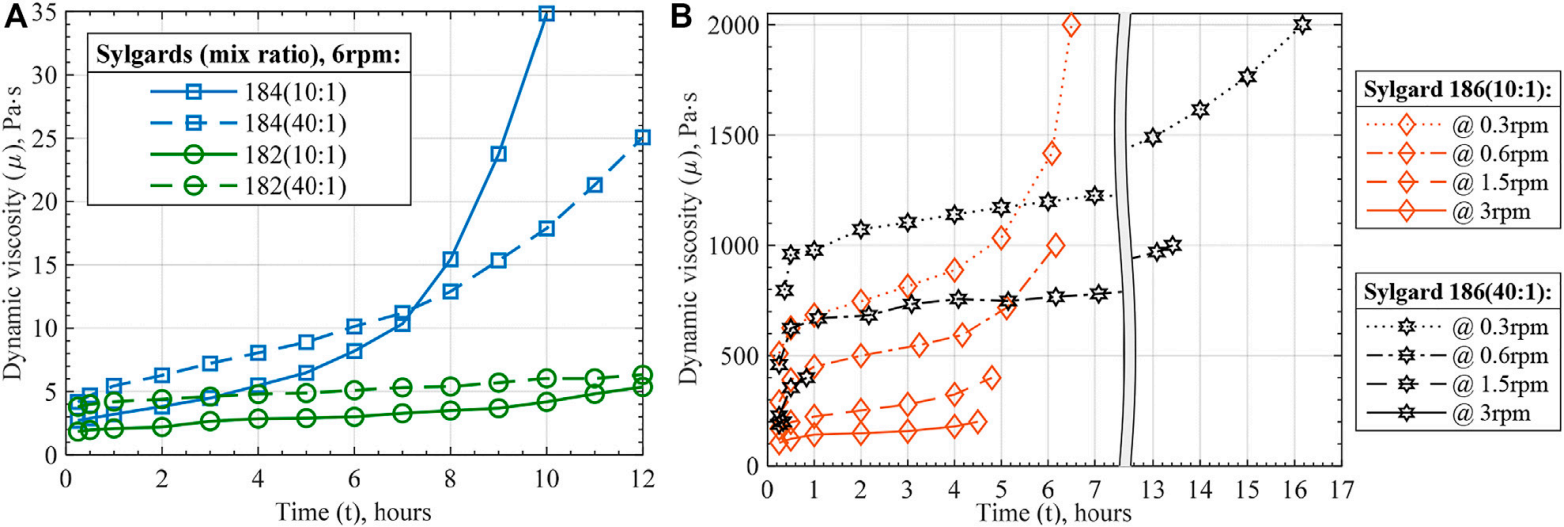
Viscosity-time profiles of the prepared silicones: **(A)** Sylgard 182 and 184, **(B)** Sylgard 186 at various viscometer revolutions per minute (rpm). The shown data points at each time instance represent average values of three to five measurements with the standard deviation of less than 1%.

The first viscosity test resulted in Figure 8 that demonstrates the change in dynamic viscosities of the materials with curing time, which can be called viscosity-time profiles. Lower initial viscosity and its steeper increase during the first fifteen to 30 minutes are attributed to the viscous heating of the silicones during the mixing process. Besides the initial region, all curves demonstrated monotonically growing viscosity and a clear exponential behavior was noticed for the fast-curing Sylgard 184 and 186 within the first 12 h.

Common for all three silicones, 40:1 ratio compositions showed higher initial viscosity. This agrees with the manufacturer’s data as higher viscosities are reported for elastomer bases than for mixed silicones. Therefore, higher content of elastomer base in 40:1 ratio composition results in a higher viscosity of the material right after the mixing. The second common characteristic is that silicones mixed in a 40:1 ratio increase their viscosity at slower rates than their 10:1 compositions. The slower rate of curing generally provides more handling time within which the materials can be processed. Nevertheless, within the 12-h study window, mixing in 40:1 ratio considerably affected the curing of Sylgard 184 and 186, but not 182. That is explained by the low rate of curing of Sylgard 182 mixed in 10:1 ratio.

Besides obtaining viscosity-curing characteristics of the mixed silicones composition, silicones' shear strain rate dependence was studied. It was noticed during the tests that Sylgard 182 and 184 viscosities have little dependence on shear strain rate. Meanwhile, Sylgard 186 demonstrated highly non-Newtonian pseudoplastic behavior (Figure 8B). Furthermore, a considerable thinning of Sylgard 186 at higher strain rates suggests that 3D printing it at higher rates eases the process wherever high viscosity reaches the equipment limitation. Particularly, the maximum strain rate was estimated to be about 0.27 1/s for 3 rpm viscometer speed and 95 1/s during the printing process of Sylgard 182 with the settings used in this work (Taylor, 1923; White and Majdalani, 2021). Lastly, the data presented in Figure 8 was used to interpolate viscosity-time functions of the Sylgard silicone compositions as for the Newtonian fluid for Sylgard 182 and 184, and the power-law fluid for Sylgard 186 (Table 2).

**TABLE 2:** Interpolated viscosity-time functions of Sylgard silicone compositions.

Sylgard silicone	Viscosity-time empirical function^a^
182 (10:1)	μ(t)=0.267t+1.64
182 (40:1)	μ(t)=0.19t+4
184 (10:1)	$\mu \left(t\right)=0.00743t^{4}-0.0674t^{3}+0.207t^{2}+0.493t+2.5$
184 (40:1)	$\mu \left(t\right)=0.0146t^{3}-0.13t^{2}+1.2t+4.36$
186 (10:1)^b^	$\mu \left(t,\dot{\gamma }\right)=\left(2.372t^{3}-15.99t^{2}+35.31t+34.95\right)∙{\dot{\gamma }}^{\left(0.002466t^{2}-0.01294t+0.3125\right)}$
186 (40:1)^b^	$\mu \left(t,\dot{\gamma }\right)=\left(3.293t+108.6\right)∙{\dot{\gamma }}^{\left(-0.001088t+0.3921\right)}$

^a^ The functions are interpolated based on time interval between 1 h and 12 h to avoid the initial silicone thinning due to mixing heating. Therefore, the functions can be used to predict the silicones’ viscosities up to the maximum time measured in the test (Figure 8).

^b^ Power-law fluid model ( $\mu_{app}=K{\dot{\gamma }}^{n-1}$ ) was used with coefficients K and n interpolated as functions of time.

The second viscosity test was conducted by printing a line pattern with time intervals until the process was interfered by the silicone polymerization (Supplementary Figure S9). The same equipment and settings were used for the test as for printing UDEAs. By observing the defects of the printed lines, particularly the dripping along the printed lines, practical handling times for the mixed silicones when printing through contact dispensing can be estimated (Table 3). The printing test results show that all 40:1 silicone compositions extended their handling time compared to the 10:1 compositions despite having larger viscosity as per the viscometer testing. The discrepancy between actual handling time and potential predictions based on the measured viscosity, can come from various manufacturing factors, e.g., the difference in strain rates and correspondingly viscosity, viscous heating, etc.

**TABLE 3:** Empirical handling time values various compositions of Sylgard silicones for contact dispensing.

Sylgard	184		186		182			
	(10:1)	(40:1)	(10:1)	(40:1)	(10:1)	(40:1)	(10:1) at 50 ℃	(10:1) at 90 ℃
Handling time for contact dispensing	6 h	9 h	4 h	6+ h^a^	11 h	12+ hours^b^	9 h	5 h

^a^ Equipment viscosity limitation have been reached hindering a higher handling time.

^b^ Test was performed up to 12 h, actual handling time is larger.

In addition, handling time of Sylgard 182 (10:1) was evaluated when printing on a heating bed at elevated temperature, and the dispensing head was raised 20 mm about the heating bed Printing at elevated temperatures showed a considerable reduction in handling time. Nevertheless, it can be used for slow-curing silicones like Sylgard 182 to speed up the printing process and minimize or completely avoid intermediate curing cycles between printing the layers.

#### 2.2.2 Elastomer electromechanical characterization

This section presents characterization of prepared silicone compositions for mechanical and dielectric properties relevant to DEA actuation. Following the discussion of each property, all the major values can be found in Table 4.

**TABLE 4:** Electromechanical characterization of the prepared PDMS compositions.

		Sylgard 184 (10:1)	Sylgard 186 (10:1)	Sylgard 182			
				10:1	20:1	30:1	40:1
Manufacturer’s Data							
Curing time at 100 ℃ , min		35	25	75	—	—	—
Tensile modulus MPa		—	—	7.3	—	—	—
Tensile strength, MPa		6.7	2.1	7.6	—	—	—
Elongation, %		—	255	105	—	—	—
Relative permittivity at 100 Hz		2.72	2.7	2.65	—	—	—
Measured							
Compressive modulus, MPa	After curing	1.15	0.864	0.957	0.272	0.095	0.0265
	After 2 months	1.09	0.86	0.842	0.238	0.12	0.03
Tensile modulus, MPa		1.25	0.774	1.31	0.634	0.0713	0.0217
Tensile strength, MPa		5.5	8	5.4	4.6	0.47	0.07
Elongation, %		200	730	280	370	515	310
Relative permittivity at 10 Hz		2.63	2.69	2.58	2.7	2.63	2.82
Breakdown strength		—	—	96.9	60.1	48.4	30.2

**FIGURE 9:**
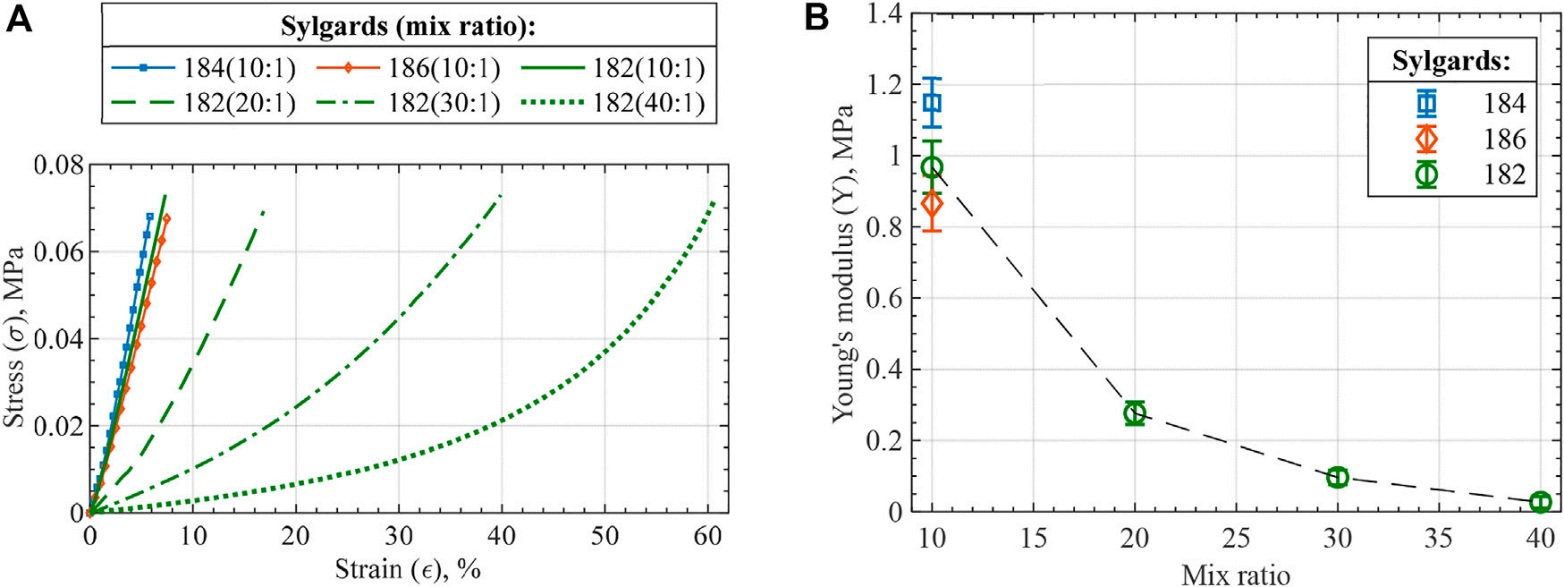
Compression test results of the prepared PDMS compositions in the form of **(A)** stress-strain curves and **(B)** Young’s modulus. The stiffness analysis of the silicones is valid at a low strain range, e.g., 0%–10%, for the performed compressive test, since the stiffening of higher mix ratio compositions is largely due to cross-section expansion.

Lowering the stiffness of DEA materials is one of the approaches to increase actuation performance, which was performed through two methods in the present study. The first method was the non-standard ratio mixing applied for PDMS Sylgard 182. The second method was curing the material at a temperature lower than stated in the manufacturer’s material data sheet but for a longer time (90 ℃ for 2 h) to ensure that no further polymerization occurs at room temperatures. As DEAs essentially operate in compressive mode when voltage is applied, compressive mechanical properties of the dielectric material are of primary interest. Figure 9A shows the obtained stress-strain curves with corresponding Young’s modulus values in Figure 9B. The first noticeable fact is the substantially more compliant behavior of PDMS Sylgard 182 mixed in higher ratios with Young’s modulus lowered by more than an order of magnitude between the lowest and highest mix ratios. Furthermore, the modulus values of silicones mixed in the standard 10:1 ratio are lower than stated by the manufacturer or in relevant studies (Vaicekauskaite et al., 2019). Particularly, silicones that has longer curing time at 100 ℃ as per manufacturer’s data were affected more by the selected curing cycle and demonstrated lower modulus values. Further materials’ polymerization at room temperature was investigated by compressive test on the same samples 2 months after curing. The results show minor modulus changes that can be partially attributed to equipment accuracy and temperature variation (Table 4). Lastly, the tensile behavior of the material was also studied with Young’s moduli and stress-strain curves presented in Table 4 and Supplementary Figure S11, respectively.

**FIGURE 10:**
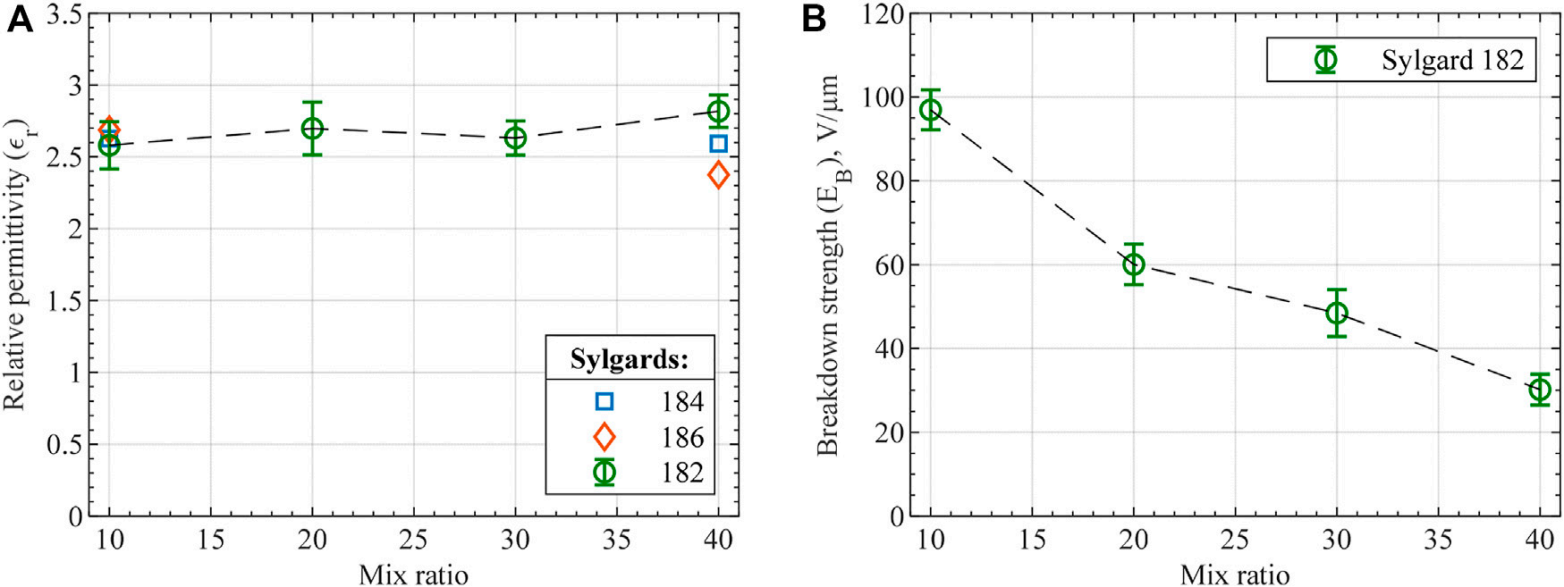
**(A)** Relative dielectric permittivity and **(B)** breakdown strength of the tested silicones. Each data point represents the average value of 5 measurements for relative permittivity and 10 measurements for breakdown strength with error bars demonstrating the standard deviation. shows Weibull probability plot for the measured breakdown strength of Sylgard 182 mix compositions.

While the silicones’ moduli are reduced, relative dielectric permittivity and dielectric breakdown strength are critical for the final actuator performance. According to the derived FOMs, relative permittivity is equally important as the modulus, while breakdown strength is squared in the FOM expressions. Thin films made of mixed silicone compositions were prepared and tested for the two dielectric properties. For Sylgard 182 samples mixed in different ratios, relative permittivity differed within the standard deviation value with a negligible ascending trend (Figure 10A). In addition to standard mix ratios, 40:1 ratio samples were tested for both Sylgard 184 and 186 to prove the minor effect of mix ratio on relative permittivity. All the relative permittivity values agree with the manufacturer’s values and show no dependency on frequency within 10^1^–10^4^ Hz range Opposite behavior was observed for the tested materials in the case of breakdown strength, as can be seen in Figure 10B. A considerable decrease in breakdown strength is apparent for larger mix ratios with a substantial property degradation between 10:1 and 20:1 mix ratios. Comparison of the obtained breakdown strength values with manufacturer’s data is complicated because the manufacturer’s values typically measured for AC voltage result in considerably smaller breakdown strength values. Nevertheless, the measured breakdown strength of 10:1 Sylgard 182 is close to similar materials across the literature (Vaicekauskaite et al., 2019; Sikulskyi et al., 2021).

#### 2.2.3 UDEA design optimization

**FIGURE 11:**
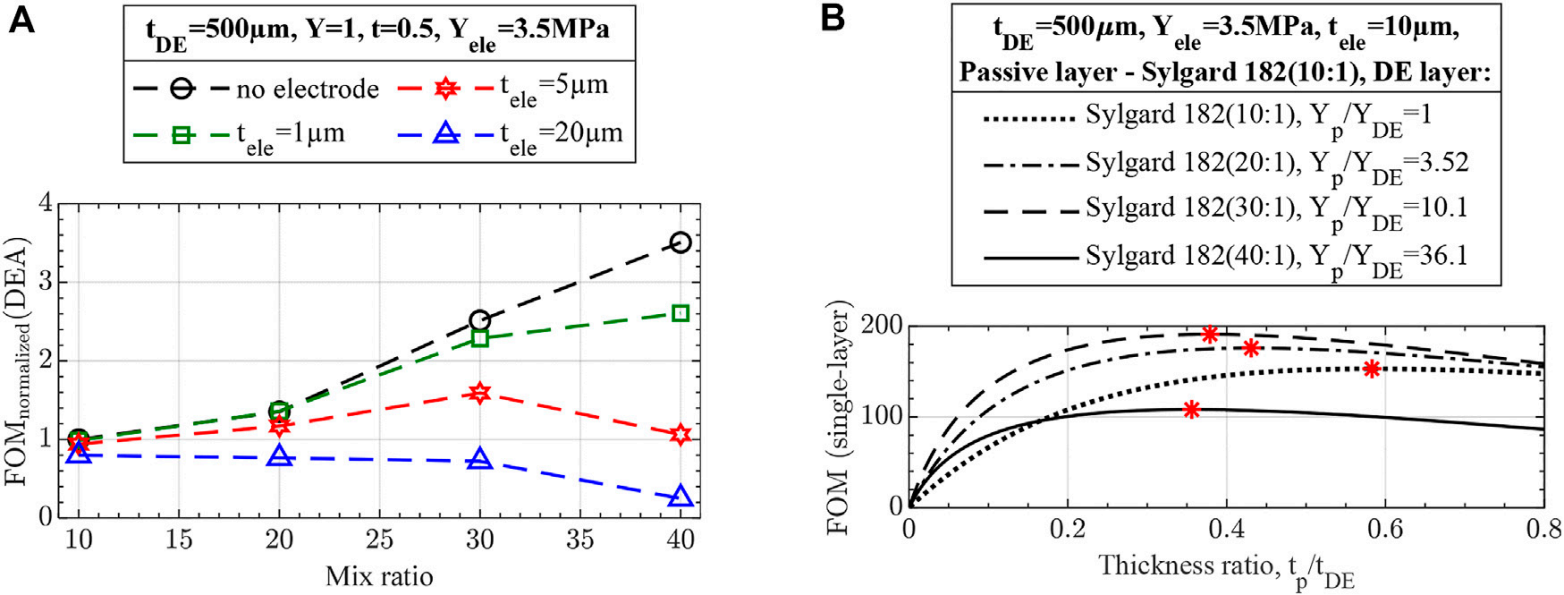
Performance of unimorph actuators through the FOM (single-layer) with **(A)** DE and passive layer sharing the same Sylgard 182 silicone mix ratio and various electrode thicknesses and **(B)** passive layer made of Sylgard 182 (10:1) ratio, various DE layers, and thinner 10 µm electrodes. Thinner electrodes were selected for the final verification not to compromise the performance enhancement of softened materials. DE layer thickness was increased to 500 µm to compensate for more compliant materials and prevent actuators’ excessive bending under their weight.

FOM is calculated for the actuator designs made of characterized materials. One option is to make both DE and passive layers of DEA out of the same mix ratio of Sylgard 182 composition. Figure 11A demonstrates the performance of the such actuator. If electrode stiffness is neglected, then the performance of the actuator increases as it is made of a more compliant Sylgard 182 composition as determined through FOM (no electrodes). However, as electrode stiffness is accounted for, the highest actuator performance can occur at silicone composition different from the most compliant one or even at the standard 10:1 mix ratio (as for the electrode used in this study).

The second design approach is a combination of mix ratios. For instance, the passive layer can be fabricated with the standard 10:1 ratio of Sylgard 182, providing a thinner and stiffer passive layer, while various mixing ratios can be evaluated as DE layers. Figure 11B shows the design plot with variable DE layer materials, passive layer thickness, and fixed electrodes used in this study. As the figure shows, the actuator with the DE layer made of 30:1 mix ratio Sylgard 182 can achieve the highest performance of about 2.5 times larger than the optimized design for the actuator entirely made of 10:1 mix ratio silicone.

#### 2.2.4 Actuation of 3D printed optimized UDEAs

**FIGURE 12:**
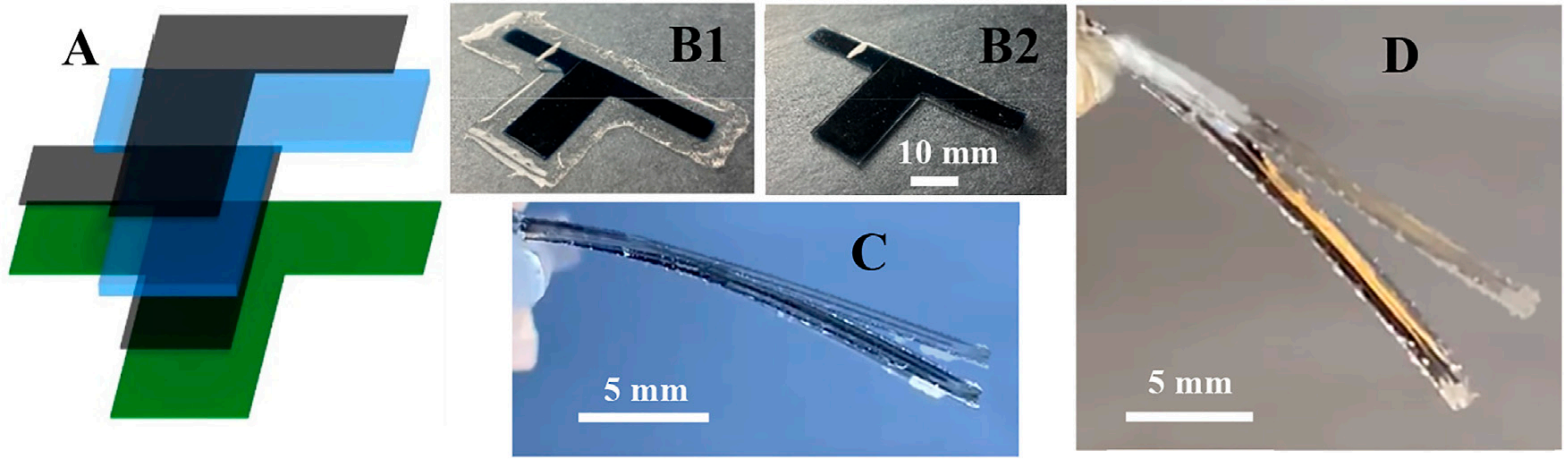
Single-layer unimorph DEA **(A)** layout, **(B1)** after printing and peeling off from the printing bed, **(B2)** after trimming and leaving some isolation material on the edges; bending of **(C)** design one due to weight only and at 9.8 kV (22.2 V/μm) applied and **(D)** design two due to weight only and at 6.4 kV (12.8 V/μm) applied.

This section presents the actuation of two single-layer UDEA designs selected based on Figure 11. The first actuator was the optimum design that was completely fabricated with Sylgard 182 (10:1) and served to validate the derived FOM for single-layer UDEA (Eq. 16). The second design was the optimized design with the passive layer fabricated with Sylgard 182 (10:1) but the DE layer made of Sylgard 182 (30:1) for achieving a higher curvature through the mixed elastomer compositions. Figure 12 demonstrates layout structure, appearance after fabrication, and actuation in the cantilever mode of both actuator designs. The design features, test results, and their comparison with analytical values are shown in Table 5. As the table shows, layers’ thicknesses of both actuators deviate from the designed values. However, the passive to DE layer thickness ratio t , is maintained resulting in near-optimum designs. The last two rows of the table validate that analytical FOM of fabricated actuators and optimum actuator designs with the same DE layer thickness are practically the same. As the experimental actuation results demonstrate, curvatures of both designs under the maximum applied voltage were predicted by the FOM with errors of about 2.5% and 4% for designs 1 and 2, respectively. The errors were attributed to uniformity and measurement of DE and passive layers’ thicknesses as well as the printed thickness of the electrodes. Design 2 proved the improved actuation capability due to the optimized design with the softened DE layer. Furthermore, the reached actuation was achieved at a considerably lower applied electric field.

**TABLE 5:** Design, experimental, and analytical performance of optimized single-layer UDEA.

		Design 1	Design 2
Designed thickness, µm (Sylgard 182 mix ratio)	DE layer	500 (10:1)	500 (30:1)
	Passive layer	341 (10:1)	214 (10:1)
	Electrode	10	10
Measured thickness for 3D printed sample, µm	DE layer	446 ± 3	521 ± 10
	Passive layer	304 ± 2	234 ± 2
	Electrode	10 ± 0.5	10 ± 0.5
Experimental actuation results	Reached electric field (E), V/µm^a^	22.2	12.8
	Reached breakdown strength ratio (E/E_B_), %^a^	22.9%	26.4%
	Curvature (k), m^−1^ (experimental FOM)	9.37	24.1
FOM (single-layer) at reached E for 3D printed designs		8.71	25.1	
FOM (single-layer) at reached E for optimized designs with the measured t_DE_ and optimum t		8.78	25.15	

^a^ Considers DEA thickness contraction during actuation.

## 3 Experimental setup

All material handling, preparation, and characterization, as well as actuator 3D printing and testing, were performed in the laboratory air environment with a temperature of 22 ± 1 ℃ and humidity of 52 ± 1%.

### 3.1 Materials

• PDMS elastomers: Sylgard 182 (Dow Inc., Midland, MI, United States, part #2065657), Sylgard 184 (Dow Inc., Midland, MI, United States, part #4019862), and Sylgard 186 (Dow Inc., Midland, MI, United States, part #2099551) are two-component temperature-cured silicones that were mixed in various ratios and tested for dielectric elastomer application and handling characteristics.
• PEDOT:PSS conductive polymer 1.1 wt.% aqueous solution (MilliporeSigma, Burlington, MA, United States, part #739332) was used as a conductive component of prepared electrode material for DEA electrodes.
• Triton X-100 plasticizer (MilliporeSigma, Burlington, MA, United States, part #X100) was used to soften PEDOT:PSS and reach sufficient compliance for the final DEA electrodes.
• Polyacrylic acid 35 wt% aqueous solution (MilliporeSigma, Burlington, MA, United States, part #416002) was used as a water soluble component of the sacrificial layer to ease the release process for thin 3D printed actuators from the printing substrate.
• Isopropanol (M.G. Chemicals Ltd., Burlington, ON, Canada, part #824) was used to further dissolve polyacrylic acid and achieve a faster evaporation rate (drying) of the sacrificial layer.

### 3.2 Material preparation

For each prepared silicone composition, the elastomer base (Part A) and curing agent (Part B) were added to the mixing cup and measured on scale to the desired weight ratio. The cup was then closed and placed into the planetary mixer THINKY ARM-310 (Laguna Hills, CA, United States) where mixing and simultaneous degassing were performed for 1 min at 2000 rpm for Sylgard 182 and 184. For Sylgard 186, an additional degassing procedure was applied due to the high viscosity of the silicone. After the initial 1-min mixing in the planetary mixer, the cup with the silicone was placed in the vacuum oven where −730 mmHg were applied for 5 min. Once the material was casted or applicated depending on the test, curing was performed at 90 ℃ for 2 h. Material characterization was performed at least 7 days after the curing to ensure residual polymerization occurs at root temperature.

For electrode material, PEDOT:PSS aqueous solution was mixed with Triton X-100 plasticizer in a ratio such that upon water evaporation, electrode consisted of 15 wt.% of PEDOT:PSS and 85 wt.% Triton X-100 (Sikulskyi, 2021). Mixing was performed with the planetary mixer THINKY ARM-310 once the materials reached a room temperature after taking them out of a fridge. The mixing cycle consisted of three steps of 5-2-5 min with the corresponding speed of 1200-2000-1200 rpm.

### 3.3 Material characterization

*Viscosity-time profile test*. Silicones were mixed and left in 150 ml plastic cups. Digital rotary viscometer NDJ-8S (KEYU, China) was used to measure the viscosity of the silicones. Switchable rotors were attached through a universal joint to the load cell of the viscometer on one end and submerged into the silicone in the cup on the other end. Rotor speed in revolution per minute (rpm) was selected according to the material viscosity and used rotor. Rotor 3 was used for Sylgards 182 and 184, and rotor 4 was used for Sylgard 186. Viscosity was measured until the limit for a specific rotor and rotational speed was reached. Curing time was counted from the moment the materials are placed in the mixer and the first measurement of the viscosity was performed at 15 min after the mixing process.

*Dielectric permittivity test*. The permittivity of the material samples was calculated based on measured capacitance $\varepsilon_{r}=\left(C/\varepsilon_{o}\right)∙\left(d/A\right)$ , where C is measured capacitance, A is electrode area, and d is the electrode spacing (dielectric film sample thickness). The measurement was performed using LCR meter GW Instek LCR-6020 (Montclair, CA, United States) and two 45 mm × 45 mm polished aluminum plates serving as electrodes on films with a thickness of about 300 µm. Appropriate electrodes dimensions and dielectric film thickness were used as per DEA testing standards ( $\sqrt{A}/d\approx 150>100$ ) (Carpi et al., 2015). The thickness of each sample was measured with disk micrometer Fowler IP54 accounting for compression during measurement, knowing the measured Young’s modulus of prepared materials and the compressive force of the micrometer of 5 N.

*Breakdown strength test*. The dielectric strength of prepared thin (100–150 µm) films was measured with a high-voltage amplifier TREK 20/20CH-S (Denver, CO, United States) by the slow rate-of-rise method according to ASTM D149. Considering the statistical nature of the breakdown, its analysis was performed based on twenty measurements for each material composition of prepared films.

*Mechanical tests*. Three tests were performed to evaluate mechanical behavior of the mixed materials. Compressive test was performed using universal test machine AMETEK CS225 (Berwyn, PA, United States) with a 5 kg load cell and compression plates G1009 on the standard ASTM D575 cylindrical specimens with a diameter of 28.2 ± 0.28 mm and height of 12.1 ± 0.8 mm (ASTM, 2018). A low compressive rate of 12 mm/min (about 100% of stain per minute) was chosen to accurately measure material’s stiffness at low strain. Compressive Young’s modulus was calculated as the slope for a relatively large strain range (0% and 5%) due to data linearity in this range and to compensate for sample imperfect shape. To prepare the specimens, mixed silicones were casted in DLP 3D printed resin molds placed on top of a PET film and cured as described above.

Second mechanical testing was evaluation of compressive behavior using Nanoindenter Bruker Hysitron TI-980 (Billerica, MA, United States). Small cylindrical samples of prepared silicones were used in the test. The force of 100 µN was applied to the 10:1 mix ratio of all three silicones, while 50 µN was applied to the 20:1 mix ratio of Sylgard 20:1 and higher mix ratios were too soft to be measured with the equipment.

Additionally, tensile test was performed to mainly evaluate effects of mix ration on stretchability and strain stiffening of silicones. Same universal test machine was used with a 1 kg load cell and wedge grip Mark-10 G1061-3 with rubber jaw faces on dogbone ASTM D412 Type D specimens cut from the 300 µm thick films with an extension rate of 40 mm/min (about 100% of strain per minute). For the tensile test, Young’s modulus was determined as the slope on the stress-strain curves between 0% and 1%.

### 3.4 3D printing of unimorph DEA

The actuators were printed using HYREL 30M (HYREL 3D, Norcross, GA, United States) printer with dispensing heads SDS-10, where elastomers and electrode material were manually loaded into the syringes after mixing and degassing. Before printing the passive layer, a glass substrate was coated with the sacrificial layer (5 wt.% polyacrylic acid, 11 wt.% water, 84 wt.% isopropanol) to ease release of thin actuators from the printing glass substrate. Before printing each electrode layer, a previously printed and cured elastomer layer underwent a plasma surface treatment with the corona surface treater ETP MODEL BD-20 (ElectroTechnic Products Inc, Chicago, IL, United States, part #12011A). To facilitate printing process, the printing bed temperature was maintained at 50 ℃ during printing. Intermediate curing of each elastomer and electrode layer took 10 min including the temperature rise from 50 ℃ to 90 ℃ . The curing of the following layers started when printing bed temperature went back to 50 ℃ . Once all layers were printed, a final curing was performed at 90 ℃ for 1 h.

### 3.5 Actuation testing

Fabricated actuators were cantilevered on one side where electrodes were attached to the high-voltage amplifier TREK 20/20CH-S. A smartphone camera was used to record the deformation of the actuators as the applied voltage was gradually and controllably increased. A custom MATLAB code was used with the recorded videos to capture actuators’ deformed shape at various values of applied voltage. The captured data was then transformed into curvature vs. electric field.

## 4 Conclusion

This study presented numerous accomplished objectives for the development of UDEAs. First, based on the derived and numerically validated analytical model, FOMs were obtained to clearly illustrate the role of each actuator layer and specific design feature effects:

• Total actuator thickness is amongst the most critical parameters to drive UDEA deformation capabilities.
• For the fixed total thickness of DEA and compliant electrode, different material combinations with optimized thickness ratios result in the same actuation performance in terms of deformation. For electrodes with finite stiffness, UDEA performance can be modified in a relatively small range based on the selected material combination of the passive and DE layers and optimized thickness ratios.
• Considering DEA 3D printing process peculiarities, fixing DE thickness rather than total thickness was recommended while optimizing the passive layer. Furthermore, different material-thickness optimum ratios were emphasized for actuators with fixed total and DE thicknesses.
• Effects of electrode thickness and modulus were demonstrated for single-layer and multilayer UDEA.
• Multilayer UDEA requires thinner DE layers to maintain actuation performance at a lower voltage (equivalent electric field). Consequently, having thin electrodes becomes even more critical to maintaining actuator performance.

Common and promising elastomer compositions were prepared, modified by varying their mixing ratios, and characterized for both actuation performance and printability objectives. For example, a considerable improvement in printing/handling time was achieved for fast-curing PDMS like Sylgard 184 and Sylgard 186 when mixed at a 40:1 ratio. Alternatively, certain mix ratios of PDMS Sylgard 182 improved their actuation performance according to the derived FOMs while already having an extensive handling/printing time.

Finally, the derived FOMs were used to obtain two different optimized UDEA designs, which were tested to validate the FOMs and demonstrate higher actuation performance of the optimized actuator fabricated with various elastomer compositions.

The future work can include considering large deflection in analytical model to accurately predict of UDEA deformation and modeling UDEA as a plate to model two-dimensional UDEA designs.

## Data Availability

The original contributions presented in the study are included in the article/Supplementary Material, further inquiries can be directed to the corresponding author.
