# Supplementary material for: Additively manufactured unimorph dielectric elastomer actuators: Design, materials, and fabrication
Source: Front Robot AI. 2022 Dec 16;9:1034914. doi: 10.3389/frobt.2022.1034914 (PMC9800877; doi:10.3389/frobt.2022.1034914)
Supplement: Supplementary file 1 [file DataSheet1.pdf]

Supplementary Material

1 Solution Process for Force and Moment Equilibrium of the Multilayer Unimorph DEA

Starting with the force equilibrium (Equation 5 in the manuscript):

$$\sum F = \sum_{i=1}^{n} b_i \int_{z_i}^{z_{i+1}} \sigma_i \, dz = \sum_{i=1}^{n} b_i Y_i \int_{z_i}^{z_{i+1}} \left(-z\kappa^\circ - \varepsilon_{DEA,i}\right) dz = 0$$

where $z_i = \bar{z} - \sum_{j=1}^{i-1} t_j$ and $z_{i+1} = \bar{z} - \sum_{j=1}^{i} t_j$.

$$\sum_{i=1}^{n} b_i Y_i \left(-\frac{z^2\kappa^\circ}{2} - z\varepsilon_{DEA,i}\right)_{z_i}^{z_{i+1}} = 0$$

$$\sum_{i=1}^{n} b_i Y_i \left(-\frac{\kappa^\circ}{2}(z_{i+1}^2 - z_i^2) - \varepsilon_{DEA,i}(z_{i+1} - z_i)\right) = 0$$

$$\sum_{i=1}^{n} b_i Y_i \left[\frac{\kappa^\circ}{2}\left(\left(\bar{z} - \sum_{j=1}^{i} t_j\right)^2 - \left(\bar{z} - \sum_{j=1}^{i-1} t_j\right)^2\right) + \varepsilon_{DEA,i}\left(\bar{z} - \sum_{j=1}^{i} t_j - \bar{z} + \sum_{j=1}^{i-1} t_j\right)\right] = 0$$

$$\sum_{i=1}^{n} b_i Y_i \left[\frac{\kappa^\circ}{2}\left(\bar{z}^2 - 2\bar{z}\sum_{j=1}^{i} t_j + \left(\sum_{j=1}^{i} t_j\right)^2 - \bar{z}^2 + 2\bar{z}\sum_{j=1}^{i-1} t_j - \left(\sum_{j=1}^{i-1} t_j\right)^2\right) - t_i\varepsilon_{DEA,i}\right] = 0$$

$$\sum_{i=1}^{n} b_i Y_i \left[\frac{\kappa^\circ}{2}\left(-2\bar{z}t_i + \left(\sum_{j=1}^{i-1} t_j + t_i\right)^2 - \left(\sum_{j=1}^{i-1} t_j\right)^2\right) - t_i\varepsilon_{DEA,i}\right] = 0$$

$$\sum_{i=1}^{n} b_i Y_i \left[\frac{\kappa^\circ}{2}\left(-2\bar{z}t_i + \left(\sum_{j=1}^{i-1} t_j\right)^2 + 2t_i\sum_{j=1}^{i-1} t_j + t_i^2 - \left(\sum_{j=1}^{i-1} t_j\right)^2\right) - t_i\varepsilon_{DEA,i}\right] = 0$$

$$\sum_{i=1}^{n} b_i Y_i \left[\frac{\kappa^\circ}{2}\left(-2\bar{z}t_i + 2t_i\sum_{j=1}^{i-1} t_j + t_i^2\right) - t_i\varepsilon_{DEA,i}\right] = 0$$

$$\sum_{i=1}^{n} b_i Y_i \left[\left(-\bar{z}\kappa^\circ t_i + \kappa^\circ t_i\sum_{j=1}^{i-1} t_j + \frac{\kappa^\circ t_i^2}{2}\right) - t_i\varepsilon_{DEA,i}\right] = 0$$

$$\kappa^\circ\bar{z}\sum_{i=1}^{n} b_i Y_i t_i = \sum_{i=1}^{n} b_i Y_i \left(\kappa^\circ t_i \sum_{j=1}^{i-1} t_j + \frac{\kappa^\circ t_i^2}{2} - t_i\varepsilon_{DEA,i}\right)$$

Solving the above for NA location, $\bar{z}$, results in the below expression (Equation 7 in the manuscript):

$$\bar{z} = \frac{\sum_{i=1}^{n} b_i Y_i t_i \left(\sum_{j=1}^{i-1} t_j + \frac{t_i}{2} - \frac{\varepsilon_{DEA,i}}{\kappa^\circ}\right)}{\sum_{i=1}^{n} b_i Y_i t_i}$$

The moment equilibrium is solved as the following (Equation 6 in the manuscript):

$$\sum M = \sum_{i=1}^{n} b_i \int_{z_i}^{z_{i+1}} \sigma_i \, zdz - \frac{\omega}{2}(L-x)^2 = 0$$

$$\sum M = \sum_{i=1}^{n} b_i \int_{z_i}^{z_{i+1}} Y_i\big(-z\kappa^\circ - \varepsilon_{DEA,i}\big) z \, dz - \frac{\omega}{2}(L-x)^2 = 0$$

$$\sum_{i=1}^{n} b_i Y_i \left(-\frac{z^3\kappa^\circ}{3} - \varepsilon_{DEA,i}\frac{z^2}{2}\right)_{z_i}^{z_{i+1}} - \frac{\omega}{2}(L-x)^2 = 0$$

$$\sum_{i=1}^{n} b_i Y_i \left(-\frac{\kappa^\circ}{3}(z_{i+1}^3 - z_i^3) - \frac{\varepsilon_{DEA,i}}{2}(z_{i+1}^2 - z_i^2)\right) - \frac{\omega}{2}(L-x)^2 = 0$$

$$\sum_{i=1}^{n} b_i Y_i \left[-\frac{\kappa^\circ}{3}\left(\left(\bar{z} - \sum_{j=1}^{i} t_j\right)^3 - \left(\bar{z} - \sum_{j=1}^{i-1} t_j\right)^3\right) - \frac{\varepsilon_{DEA,i}}{2}\left(\left(\bar{z} - \sum_{j=1}^{i} t_j\right)^2 - \left(\bar{z} - \sum_{j=1}^{i-1} t_j\right)^2\right)\right] - \frac{\omega}{2}(L-x)^2 = 0$$

$$\sum_{i=1}^{n} b_i Y_i \left[-\frac{\kappa^\circ}{3}\left(\bar{z}^3 - 3\bar{z}^2\sum_{j=1}^{i} t_j + 3\bar{z}\left(\sum_{j=1}^{i} t_j\right)^2 - \left(\sum_{j=1}^{i} t_j\right)^3 - \bar{z}^3 + 3\bar{z}^2\sum_{j=1}^{i-1} t_j - 3\bar{z}\left(\sum_{j=1}^{i-1} t_j\right)^2 + \left(\sum_{j=1}^{i-1} t_j\right)^3\right)\right.$$
$$\left. - \frac{\varepsilon_{DEA,i}}{2}\left(\bar{z}^2 - 2\bar{z}\sum_{j=1}^{i} t_j + \left(\sum_{j=1}^{i} t_j\right)^2 - \bar{z}^2 + 2\bar{z}\sum_{j=1}^{i-1} t_j - \left(\sum_{j=1}^{i-1} t_j\right)^2\right)\right] - \frac{\omega}{2}(L-x)^2 = 0$$

$$\sum_{i=1}^{n} b_i Y_i \left[\kappa^\circ t_i\left(\bar{z}^2 - \bar{z}\left(2\sum_{j=1}^{i-1} t_j + t_i\right) + \left(\sum_{j=1}^{i-1} t_j\right)^2 + t_i\sum_{j=1}^{i-1} t_j + \frac{t_i^2}{3}\right) + t_i\varepsilon_{DEA,i}\left(\bar{z} - \sum_{j=1}^{i-1} t_j - \frac{t_i}{2}\right)\right] - \frac{\omega}{2}(L-x)^2 = 0$$

Expressing curvature κ° from above results in the final expression (Equation 9 in the manuscript):

$$\kappa^\circ = \frac{\frac{\omega}{2}(L-x)^2 + \sum_{i=1}^{n} b_i Y_i t_i \varepsilon_{DEA,i}\left(\sum_{j=1}^{i-1} t_j + \frac{t_i}{2} - \bar{z}\right)}{\sum_{i=1}^{n} b_i Y_i t_i\left(\left(\sum_{j=1}^{i-1} t_j\right)^2 + t_i\sum_{j=1}^{i-1} t_j + \frac{t_i^2}{3} + \bar{z}^2 - \bar{z}\left(2\sum_{j=1}^{i-1} t_j + t_i\right)\right)}$$

2 Discussion of the small deflection analytical model

The above equations can be used to find actuator's NA location and curvature, and corresponding deflection. When analyzing a cantilever single-layer unimorph DEA (one passive layer and one DE layer between two electrodes), several things can be noticed relative to its deflection and NA:

- When no voltage is applied but weight is considered, the unimorph actuator will bend with a variable curvature and a constant NA location that depends on the multilayer structure of the beam
- When voltage is applied to DEA and weight is not considered, a unimorph DEA will bend with a constant curvature and NA location dependent on the applied voltage
- When both weight and DEA actuation are considered, both curvature and NA location will change along the length of the beam

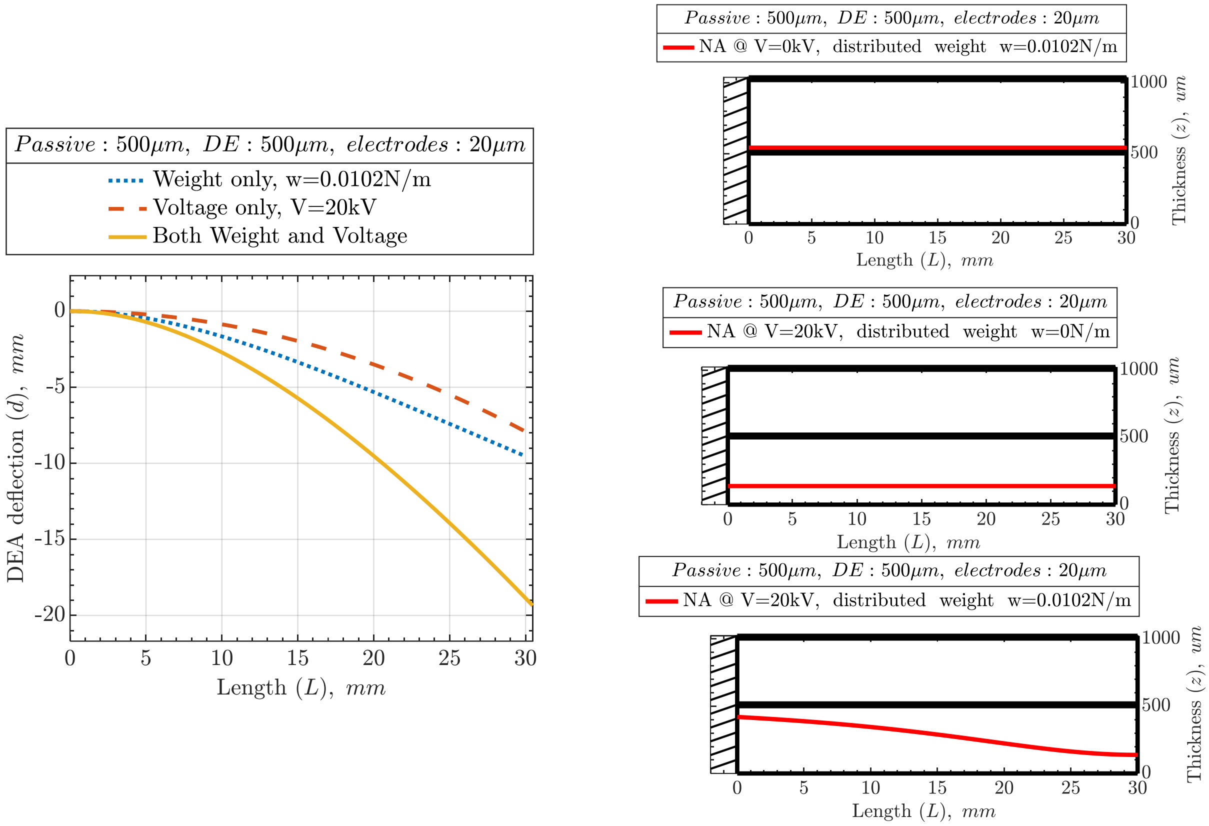

Supplementary Figure 1. Deflection and NA location comparison for different load cases of a cantilever single-layer unimorph DEA using the derived small deflection analytical model.

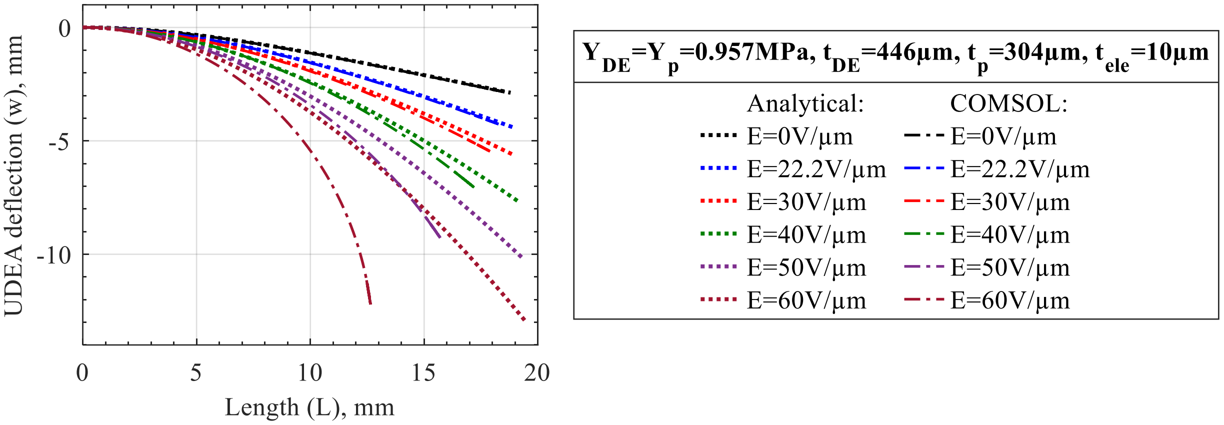

Supplementary Figure 2. Analytically and numerically calculated UDEA deformed shapes at various applied electric fields. Electric field of 22.2 V/µm corresponds to the maximum electric field applied in the experiment. Further values of electric field are gradually increase up to the value of 60 V/µm that approximately corresponds to the maximum value reaching for 3D printed DEA in the literature.

3 Solution Process for Figure of Merit

Derivation starts from the curvature Equations 7 and 8 with the following assumptions as discussed in the main manuscript:

1. Actuator's weight can be neglected because the objective of FOM is to achieve maximum actuation.
2. Cross-section is rectangular with width, b, same across passive, electrode, and DE layers, i.e., neglecting the isolation material:

$$\kappa^{\circ} = \frac{\sum_{i=1}^{n} Y_i t_i\, \varepsilon_{DEA,i}}{\sum_{i=1}^{n} Y_i t_i \left(\sum_{j=1}^{i-1} t_j + \frac{t_i}{2} - \bar{z}\right)}$$

$$\kappa^{\circ} = \frac{\sum_{i=1}^{n} Y_i t_i \varepsilon_{DEA,i} \left(\sum_{j=1}^{i-1} t_j + \frac{t_i}{2} - \bar{z}\right)}{\sum_{i=1}^{n} Y_i t_i \left(\left(\sum_{j=1}^{i-1} t_j\right)^2 + t_i \sum_{j=1}^{i-1} t_j + \frac{t_i^2}{3} + \bar{z}^2 - \bar{z}\left(2\sum_{j=1}^{i-1} t_j + t_i\right)\right)}$$

3. A typical unimorph material layout starts with the passive layer and then alternates between electrode and DE layers ($n_p = 1, n_{ele} = n_{DE} + 1$).
4. All DE layers have the same thickness and all electrodes have the same thickness.
5. Same voltage potential difference (voltage) is applied to all electrodes.
6. Actuation strain, $\varepsilon_{DEA,i}$, happens in DE layers only.

$$\kappa^{\circ} = \frac{n_{DE} Y_{DE} t_{DE} \varepsilon_{DEA}}{Y_p t_p \left(\frac{t_p}{2} - \bar{z}\right) + Y_{DE} t_{DE} \sum_{i=1}^{n_{DE}} \left(\left(t_p + (i-1)t_{DE} + i t_{ele}\right) + \frac{1}{2} t_{DE} - \bar{z}\right) + Y_{ele} t_{ele} \sum_{i=1}^{n_{DE}+1} \left(\left(t_p + (i-1)t_{DE} + (i-1)t_{ele}\right) + \frac{1}{2} t_{ele} - \bar{z}\right)}$$

$$\kappa^{\circ} = \frac{n_{DE} Y_{DE} t_{DE} \varepsilon_{DEA}}{Y_p t_p \left(\frac{t_p}{2} - \bar{z}\right) + Y_{DE} t_{DE} \left(\left(n_{DE} t_p + \frac{n_{DE}(n_{DE}-1)t_{DE}}{2} + \frac{n_{DE}(n_{DE}+1)t_{ele}}{2}\right) + \frac{n_{DE}}{2} t_{DE} - n_{DE}\bar{z}\right) + Y_{ele} t_{ele} \left(\left((n_{DE}+1)t_p + \frac{n_{DE}(n_{DE}+1)t_{DE}}{2} + \frac{n_{DE}(n_{DE}+1)t_{ele}}{2}\right) + \frac{(n_{DE}+1)}{2} t_{ele} - (n_{DE}+1)\bar{z}\right)}$$

$$\kappa^{\circ} = \frac{n_{DE} Y_{DE} t_{DE} \varepsilon_{DEA}}{Y_p t_p \left(\frac{t_p}{2} - \bar{z}\right) + Y_{DE} t_{DE} n_{DE} \left(t_p + \frac{n_{DE} t_{DE}}{2} + \frac{(n_{DE}+1)t_{ele}}{2} - \bar{z}\right) + Y_{ele} t_{ele} (n_{DE}+1) \left(t_p + \frac{n_{DE} t_{DE}}{2} + \frac{(n_{DE}+1)t_{ele}}{2} - \bar{z}\right)}$$

Second curvature equation obtained from the moment equilibrium is simplified as the following:

$$\kappa^{\circ} = \frac{Y_{DE} t_{DE} \varepsilon_{DEA} \sum_{i=1}^{n} \left(\left(t_p + (i-1)t_{DE} + i t_{ele}\right) + \frac{t_{DE}}{2} - \bar{z}\right)}{\sum_{i=1}^{n} Y_i t_i \left(\left(\sum_{j=1}^{i-1} t_j\right)^2 + t_i \sum_{j=1}^{i-1} t_j + \frac{t_i^2}{3} + \bar{z}^2 - \bar{z}\left(2\sum_{j=1}^{i-1} t_j + t_i\right)\right)}$$

$$\kappa^{\circ} = \frac{Y_{DE} t_{DE} \varepsilon_{DEA} \sum_{i=1}^{n} \left(\left(t_p + (i-1)t_{DE} + i t_{ele}\right) + \frac{t_{DE}}{2} - \bar{z}\right)}{Y_p t_p \left(\frac{t_p^2}{3} + \bar{z}^2 - \bar{z} t_p\right) + Y_{DE} t_{DE} C_{DE} + Y_{ele} t_{ele} C_{ele}}$$

$$C_{DE} = \sum_{i=1}^{n} \left(\left(t_p + (i-1)t_{DE} + i t_{ele}\right)^2 + t_{DE}\left(t_p + (i-1)t_{DE} + i t_{ele}\right) + \frac{t_{DE}^2}{3} + \bar{z}^2 - \bar{z}\left(2\left(t_p + (i-1)t_{DE} + i t_{ele}\right) + t_{DE}\right)\right)$$

$$C_{ele} = \sum_{i=1}^{n+1} \left(\left(t_p + (i-1)t_{DE} + (i-1)t_{ele}\right)^2 + t_{ele}\left(t_p + (i-1)t_{DE} + (i-1)t_{ele}\right) + \frac{t_{ele}^2}{3} + \bar{z}^2 - \bar{z}\left(2\left(t_p + (i-1)t_{DE} + (i-1)t_{ele}\right) + t_{ele}\right)\right)$$

$$\kappa^{\circ} = \frac{\varepsilon_{DEA} Y_{DE} t_{DE} n_{DE} \left(t_p + \frac{n_{DE} t_{DE}}{2} + \frac{(n_{DE}+1)t_{ele}}{2} - \bar{z}\right)}{Y_p t_p \left(\frac{t_p^2}{3} + \bar{z}^2 - \bar{z} t_p\right) + Y_{DE} t_{DE} n_{DE} A + Y_{ele} t_{ele} (n_{DE}+1) B}$$

where:

$$A = t_p^2 + \frac{1}{3} n_{DE}^2 t_{DE}^2 + \frac{1}{6}(2n_{DE}^2 + 3n_{DE} + 1)t_{ele}^2 + n_{DE} t_{DE} t_p + (n_{DE}+1) t_p t_{ele} + \frac{1}{6}(4n_{DE}^2 + 3n_{DE} - 1) t_{DE} t_{ele} + \bar{z}^2 - 2\bar{z}\left(t_p + \frac{n_{DE} t_{DE}}{2} + \frac{(n_{DE}+1)t_{ele}}{2}\right)$$

$$B = t_p^2 + \frac{1}{6}(2n_{DE}^2 + n_{DE})(t_{DE} + t_{ele})^2 + \left(\frac{n_{DE}}{2} + \frac{1}{3}\right) t_{ele}^2 + n_{DE} t_{DE} t_p + \frac{1}{2} n_{DE} t_{DE} t_{ele} + (n_{DE}+1) t_{ele} t_p + \bar{z}^2 - \bar{z}\left(2t_p + t_{ele} + n_{DE}(t_{DE} + t_{ele})\right)$$

Derivation of A and B values:

$$\begin{aligned}
&\sum_{i=1}^{n_{DE}}\left(t_p+(i-1)t_{DE}+it_{ele}\right)^2+\sum_{i=1}^{n_{DE}}t_{DE}\left(t_p+(i-1)t_{DE}+it_{ele}\right)+n_{DE}\frac{t_{DE}^2}{3}+n_{DE}\bar{z}^2-\sum_{i=1}^{n_{DE}}\bar{z}\left(2\left(t_p+(i-1)t_{DE}+it_{ele}\right)+t_{DE}\right)\\
&=\sum_{i=1}^{n_{DE}}\left(t_p^2+(i^2-2i+1)t_{DE}^2+(it_{ele})^2+2\left((i-1)t_{DE}t_p+it_pt_{ele}+(i^2-i)t_{DE}t_{ele}\right)\right)+t_{DE}\sum_{i=1}^{n_{DE}}\left(t_p+(i-1)t_{DE}+it_{ele}\right)+n_{DE}\frac{t_{DE}^2}{3}+n_{DE}\bar{z}^2\\
&-2\bar{z}\sum_{i=1}^{n_{DE}}\left(\left(t_p+(i-1)t_{DE}+it_{ele}\right)+\frac{t_{DE}}{2}\right)\\
&=\left(n_{DE}t_p^2+\left(\frac{n_{DE}}{6}(2n_{DE}^2+3n_{DE}+1)-n_{DE}(n_{DE}+1)+n_{DE}\right)t_{DE}^2+\frac{n_{DE}}{6}(2n_{DE}^2+3n_{DE}+1)t_{ele}^2\right.\\
&\left.+2\left(\frac{n_{DE}(n_{DE}-1)t_{DE}t_p}{2}+\frac{n_{DE}(n_{DE}+1)}{2}t_pt_{ele}+\left(\frac{n_{DE}}{6}(2n_{DE}^2+3n_{DE}+1)-\frac{n_{DE}(n_{DE}+1)}{2}\right)t_{DE}t_{ele}\right)\right)+t_{DE}\left(n_{DE}t_p+\frac{n_{DE}(n_{DE}-1)t_{DE}}{2}+\frac{n_{DE}(n_{DE}+1)t_{ele}}{2}\right)+n_{DE}\frac{t_{DE}^2}{3}\\
&+n_{DE}\bar{z}^2-2\bar{z}\left(n_{DE}t_p+\frac{n_{DE}(n_{DE}-1)t_{DE}}{2}+\frac{n_{DE}(n_{DE}+1)t_{ele}}{2}+\frac{n_{DE}t_{DE}}{2}\right)\\
&=n_{DE}\left[t_p^2+\frac{1}{6}(2n_{DE}^2-3n_{DE}+1)t_{DE}^2+\frac{1}{6}(2n_{DE}^2+3n_{DE}+1)t_{ele}^2+2\left(\frac{(n_{DE}-1)t_{DE}t_p}{2}+\frac{(n_{DE}+1)}{2}t_pt_{ele}+\frac{1}{6}(2n_{DE}^2-2)t_{DE}t_{ele}\right)\right.\\
&\left.+t_{DE}\left(t_p+\frac{(n_{DE}-1)t_{DE}}{2}+\frac{(n_{DE}+1)t_{ele}}{2}\right)+\frac{t_{DE}^2}{3}+\bar{z}^2-2\bar{z}\left(t_p+\frac{n_{DE}t_{DE}}{2}+\frac{(n_{DE}+1)t_{ele}}{2}\right)\right]\\
&=n_{DE}\left[t_p^2+\frac{1}{3}n_{DE}^2t_{DE}^2+\frac{1}{6}(2n_{DE}^2+3n_{DE}+1)t_{ele}^2+n_{DE}t_{DE}t_p+(n_{DE}+1)t_pt_{ele}+\frac{1}{6}(4n_{DE}^2+3n_{DE}-1)t_{DE}t_{ele}+\bar{z}^2-2\bar{z}\left(t_p+\frac{n_{DE}t_{DE}}{2}+\frac{(n_{DE}+1)t_{ele}}{2}\right)\right]=n_{DE}A
\end{aligned}$$

$$\begin{aligned}
&\sum_{i=1}^{n_{DE}+1}\left(t_p+(i-1)t_{DE}+(i-1)t_{ele}\right)^2+\sum_{i=1}^{n_{DE}+1}t_{ele}\left(t_p+(i-1)t_{DE}+(i-1)t_{ele}\right)+(n_{DE}+1)\frac{t_{ele}^2}{3}+(n_{DE}+1)\bar{z}^2-\sum_{i=1}^{n_{DE}+1}\bar{z}\left(2\left(t_p+(i-1)t_{DE}+(i-1)t_{ele}\right)+t_{ele}\right)\\
&=\sum_{i=1}^{n_{DE}+1}\left(t_p^2+(i^2-2i+1)(t_{DE}^2+{t_{ele}}^2)+2\left((i-1)(t_{DE}+t_{ele})t_p+(i^2-2i+1)t_{DE}t_{ele}\right)\right)+t_{ele}\sum_{i=1}^{n_{DE}+1}\left(t_p+(i-1)(t_{DE}+t_{ele})\right)+(n_{DE}+1)\frac{t_{ele}^2}{3}+(n_{DE}+1)\bar{z}^2\\
&-2\bar{z}\sum_{i=1}^{n_{DE}+1}\left(\left(t_p+(i-1)(t_{DE}+t_{ele})\right)+\frac{t_{ele}}{2}\right)\\
&=\left((n_{DE}+1)t_p^2+\left(\frac{(n_{DE}+1)}{6}(2n_{DE}^2+7n_{DE}+6)-(n_{DE}+1)(n_{DE}+2)+(n_{DE}+1)\right)(t_{DE}^2+t_{ele}^2)\right.\\
&\left.+2\left(\frac{n_{DE}(n_{DE}+1)}{2}(t_{DE}+t_{ele})t_p+\left(\frac{(n_{DE}+1)}{6}(2n_{DE}^2+7n_{DE}+6)-(n_{DE}+1)(n_{DE}+2)+(n_{DE}+1)\right)t_{DE}t_{ele}\right)\right)+t_{ele}\left((n_{DE}+1)t_p+\frac{n_{DE}(n_{DE}+1)}{2}(t_{DE}+t_{ele})\right)\\
&+(n_{DE}+1)\frac{t_{ele}^2}{3}+(n_{DE}+1)\bar{z}^2-2\bar{z}\left((n_{DE}+1)t_p+\frac{n_{DE}(n_{DE}+1)}{2}(t_{DE}+t_{ele})+\frac{(n_{DE}+1)t_{ele}}{2}\right)\\
&=(n_{DE}+1)\left[t_p^2+\frac{1}{6}(2n_{DE}^2+n_{DE})(t_{DE}^2+t_{ele}^2)+n_{DE}(t_{DE}+t_{ele})t_p+\frac{1}{3}(2n_{DE}^2+n_{DE})t_{DE}t_{ele}+t_{ele}\left(t_p+\frac{n_{DE}}{2}(t_{DE}+t_{ele})\right)+\frac{t_{ele}^2}{3}+\bar{z}^2-\bar{z}\left(2t_p+n_{DE}(t_{DE}+t_{ele})+t_{ele}\right)\right]\\
&=(n_{DE}+1)\left[t_p^2+\frac{1}{6}(2n_{DE}^2+n_{DE})(t_{DE}^2+2t_{DE}t_{ele}+t_{ele}^2)+\frac{t_{ele}^2}{3}+n_{DE}(t_{DE}+t_{ele})\left(t_p+\frac{t_{ele}}{2}\right)+t_{ele}t_p+\bar{z}^2-\bar{z}\left(2t_p+t_{ele}+n_{DE}(t_{DE}+t_{ele})\right)\right]\\
&=(n_{DE}+1)\left[t_p^2+\frac{1}{6}(2n_{DE}^2+n_{DE})(t_{DE}+t_{ele})^2+\left(\frac{n_{DE}}{2}+\frac{1}{3}\right)t_{ele}^2+n_{DE}t_{DE}t_p+\frac{1}{2}n_{DE}t_{DE}t_{ele}+(n_{DE}+1)t_{ele}t_p+\bar{z}^2-\bar{z}\left(2t_p+t_{ele}+n_{DE}(t_{DE}+t_{ele})\right)\right]=(n_{DE}+1)B
\end{aligned}$$

7. If $n_{DE}=1$, the first curvature equation obtained from the force equilibrium is simplified as the following:

$$\kappa^{\circ}=\frac{Y_{DE}t_{DE}\varepsilon_{DEA}}{Y_pt_p\left(\frac{t_p}{2}-\bar{z}\right)+(Y_{DE}t_{DE}+2Y_{ele}t_{ele})\left(t_p+\frac{t_{DE}}{2}+t_{ele}-\bar{z}\right)}$$

The second curvature equation obtained from the moment equilibrium is simplified as the following:

$$\kappa^{\circ}=\frac{\varepsilon_{DEA}Y_{DE}t_{DE}\left(t_p+\frac{t_{DE}}{2}+t_{ele}-\bar{z}\right)}{Y_pt_p\left(\frac{t_p^2}{3}+\bar{z}^2-\bar{z}t_p\right)+Y_{DE}t_{DE}A+2Y_{ele}t_{ele}B}$$

Where

$$A=t_p^2+\frac{1}{3}t_{DE}^2+t_{ele}^2+t_{DE}t_p+2t_pt_{ele}+t_{DE}t_{ele}+\bar{z}^2-2\bar{z}\left(t_p+\frac{t_{DE}}{2}+t_{ele}\right)$$

$$B=t_p^2+\frac{1}{2}(t_{DE}+t_{ele})^2+\frac{5}{6}t_{ele}^2+t_{DE}t_p+\frac{1}{2}t_{DE}t_{ele}+2t_{ele}t_p+\bar{z}^2-2\bar{z}\left(t_p+\frac{t_{DE}}{2}+t_{ele}\right)$$

The above equations can already result in the explicit curvature expression

$$k=\frac{6\varepsilon_{DEA}Y_{DE}Y_pt_{DE}t_p\left(t_{DE}+t_p+2t_{ele}\right)}{Y_{DE}^2t_{DE}^4+Y_p^2t_p^4+Y_{DE}Y_{ele}t_{DE}t_{ele}(8t_{DE}^2+12t_{DE}t_{ele}+8t_{ele}^2)+Y_{DE}Y_pt_{DE}t_p\left(4t_{DE}^2+12t_{DE}t_{ele}+6t_{DE}t_p+12t_{ele}^2+12t_pt_{ele}+4t_p^2\right)+Y_{ele}^2t_{ele}^2(12t_{DE}^2+24t_{DE}t_{ele}+16t_{ele}^2)+Y_{ele}Y_pt_{ele}t_p\left(12t_{DE}^2+36t_{DE}t_{ele}+12t_{DE}t_p+32t_{ele}^2+24t_pt_{ele}+8t_p^2\right)}$$

Divide nominator and denominator by $(Y_{DE}^2 t_{DE}^3)$ and let $Y = Y_p/Y_{DE}$ and $t = t_p/t_{DE}$:

$$\boldsymbol{k} = \frac{6\varepsilon_{DEA} Y\left(t + t^2 + 2t\frac{t_{ele}}{t_{DE}}\right)}{t_{DE} + Y^2 t_p t^3 + \frac{Y_{ele}}{Y_{DE}} t_{ele}\left(8 + 12\frac{t_{ele}}{t_{DE}} + 8\frac{t_{ele}^2}{t_{DE}^2}\right) + Yt_p\left(4 + 12\frac{t_{ele}}{t_{DE}} + 6t + 12\frac{t_{ele}^2}{t_{DE}^2} + 12t\frac{t_{ele}}{t_{DE}} + 4t^2\right) + \frac{Y_{ele}^2}{Y_{DE}^2}\frac{t_{ele}^2}{t_{DE}}\left(12 + 24\frac{t_{ele}}{t_{DE}} + 16\frac{t_{ele}^2}{t_{DE}^2}\right) + \frac{Y_{ele}}{Y_{DE}} Ytt_{ele}\left(12 + 36\frac{t_{ele}}{t_{DE}} + 12t + 32\frac{t_{ele}^2}{t_{DE}^2} + 24t\frac{t_{ele}}{t_{DE}} + 8t^2\right)}$$

Let $R_Y = Y_{ele}/Y_{DE}$ and $R_t = t_{ele}/t_{DE}$:

$$k = \frac{6\varepsilon_{DEA} Y(t + t^2 + 2tR_t)}{t_{DE} + Y^2 t_p t^3 + R_Y t_{ele}(8 + 12R_t + 8R_t^2) + Yt_p(4 + 12R_t + 6t + 12R_t^2 + 12tR_t + 4t^2) + R_Y^2 R_t t_{ele}(12 + 24R_t + 16R_t^2) + R_Y Ytt_{ele}(12 + 36R_t + 12t + 32R_t^2 + 24tR_t + 8t^2)}$$

$$FOM\ (single - layer) = \frac{6\boldsymbol{\mu\varepsilon_r\varepsilon_o E_B^2} Y(t + t^2 + 2tR_t)}{Y_{DE} t_{DE}\left(1 + Y^2 t^4 + R_Y R_t(8 + 12R_t + 8R_t^2) + Yt(4 + 12R_t + 6t + 12R_t^2 + 12tR_t + 4t^2) + R_Y^2 R_t^2(12 + 24R_t + 16R_t^2) + R_Y R_t Yt(12 + 36R_t + 12t + 32R_t^2 + 24tR_t + 8t^2)\right)}$$

8.1. If $n_{DE} = 1$ & $Y_{ele} = 0$ (same assumptions as for planar DEA FOM, but still accounting for the electrode thickness because it contributes to the unimorph actuator's total thickness):

$$FOM\ (Y_{ele} = 0) = k = \frac{6\mu\varepsilon_r\varepsilon_o E_B^2 Y(t + t^2 + 2tR_t)}{Y_{DE} t_{DE}\left(1 + Y^2 t^4 + Yt(4 + 12R_t + 6t + 12R_t^2 + 12tR_t + 4t^2)\right)}$$

8.2. If $n_{DE} = 1$ & $Y_{ele} = 0$ & $t_{ele} = 0$ (for very thin electrodes compared to DE and passive layers):

$$FOM\ (no\ electrode) = k = \frac{6\mu\varepsilon_r\varepsilon_o E_B^2 Y(t + t^2)}{Y_{DE} t_{DE}\left(1 + Y^2 t^4 + Yt(4 + 6t + 4t^2)\right)}$$

3.1 Derivation of the optimized unimorph DEA design

For the above FOM (no electrode) expression, optimum modulus-thickness ratios $(Y - t)$ can be found:

$$\frac{d(FOM)}{dY} = 0 = \frac{6\mu\varepsilon_r\varepsilon_o E_B^2(t + t^2)}{Y_{DE} t_{DE}}\left(\frac{1}{D} - \frac{Y(t(4t^2 + 6t + 4) + 2Yt^4)}{D^2}\right)$$

where $D = Y^2 t^4 + Yt(4t^2 + 6t + 4) + 1$

The first multiplier cannot be zero, therefore:

$$\left(\frac{1}{D} - \frac{Y(t(4t^2 + 6t + 4) + 2Yt^4)}{D^2}\right) = 0 => Y^2 t^4 + Yt(4t^2 + 6t + 4) + 1 - Y(t(4t^2 + 6t + 4) + 2Yt^4) = 1 - Y^2 t^4$$

Then, $t = \frac{1}{\sqrt{Y}}$ is the optimum modulus-thickness ration for UDEA with the fixed total thickness and found through the no-electrode FOM.

Alternatively:

$$\frac{d(FOM)}{dt} = 0 = \frac{6\mu\varepsilon_r\varepsilon_o E_B^2 Y}{Y_{DE} t_{DE}}\left(\frac{2t + 1}{D} - \frac{(t^2 + t)\left(Y(4t^2 + 6t + 4) + 4Y^2 t^3 + Yt(8t + 6)\right)}{D^2}\right)$$

Similarly, the first multiplier cannot be zero, therefore:

$$\left(\frac{2t + 1}{D} - \frac{(t^2 + t)\left(Y(4t^2 + 6t + 4) + 4Y^2 t^3 + Yt(8t + 6)\right)}{D^2}\right) = 0$$

$$(2t + 1)(Y^2 t^4 + Yt(4t^2 + 6t + 4) + 1) - (t^2 + t)\left(Y(4t^2 + 6t + 4) + 4Y^2 t^3 + Yt(8t + 6)\right) = 0$$

After simplifying:

$$2Y^2t^5 + 3Y^2t^4 + 2Yt^2(2t^2 + 4t + 1) - 2t - 1 = 0$$

Solve considering $Y > 0$ and $t > 0$:

$$Y = \frac{1}{t^2(2t + 3)}$$

3.2 Optimized Unimorph DEA Bending under Weight

For the experimentation and practical application purposes, UDEA bending under its weight is of interest. Therefore, the optimized designs are evaluated using the derived analytical model for their bending under the weight only, i.e., no voltage applied (Supplementary Figure 3).

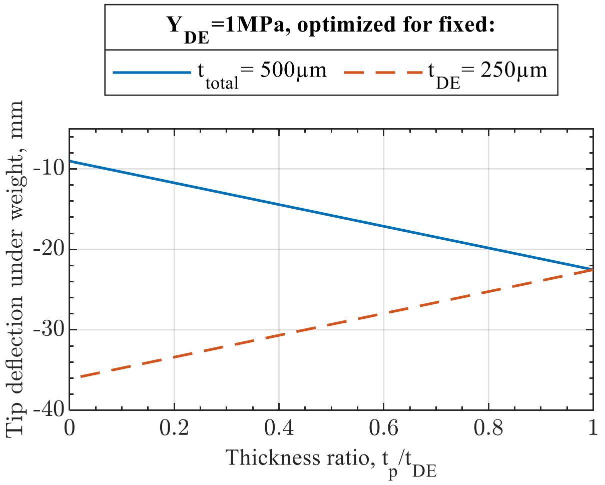

Supplementary Figure 3. Tip deflections of unimorph DEA designs optimized for fixed t_{total} and t_{DE} (for each thickness ratio, modulus are optimized using $t = \frac{1}{\sqrt{Y}}$ and $Y = \frac{1}{t^2(2t+3)}$ for fixed t_{total} and t_{DE}, respectively). Densities of both passive and DE layers are assumed to be 1 gr/cm^3.

3.3 Effect of Electrodes on Unimorph DEA Actuation

Essentially, electrodes' effects consist of the increased total actuator thickness and the actual electrodes' stiffness. Cumulative effect of components and individual contribution of the increased thickness are analyzed in the manuscript of the paper. Here, the individual effect of electrodes' stiffness is analyzed.

Individual effect of electrodes' stiffness excluding the increased total actuator thickness can be evaluated by fixing the total thickness. Supplemental Figure 4a shows how the selected electrodes' thickness and modulus affect the actuator with fixed total thickness. The effect of electrodes on the actuator with fixed total thickness is similar but smaller than the effect on the actuator with fixed DE layer (Figure 5). Particularly, actuation performance of the actuators with thin and stiff passive layers is compromised less by adding electrodes than the performance of actuators with thicker and softer passive layer. It is explained by a larger actuator stiffness for the optimized designs with thinner and stiffer passive layers as can be seen from unimorph actuator deflection under its weight (Supplemental Figure 4). As a result, added electrode stiffness contribute relatively less to the initially stiffer optimized actuator with thin and stiff passive layer. Nevertheless, fixing total thickness and adding electrodes means that thicknesses of both passive and DE layers must be decreased which might negatively affect printing quality of the films, particularly DE layer. To further illustrate electrode effects on the fixed total thickness UDEA, Supplemental Figure 4b shows FOM for various electrode thickness ratios, R_t, and fixed R_Y. For an actuator with the fixed total thickness, electrode with very low modulus can improve actuation merely by reducing overall actuator stiffness. However, for most cases the performance of actuators with fixed thickness is degraded considerably as electrodes' thickness increases.

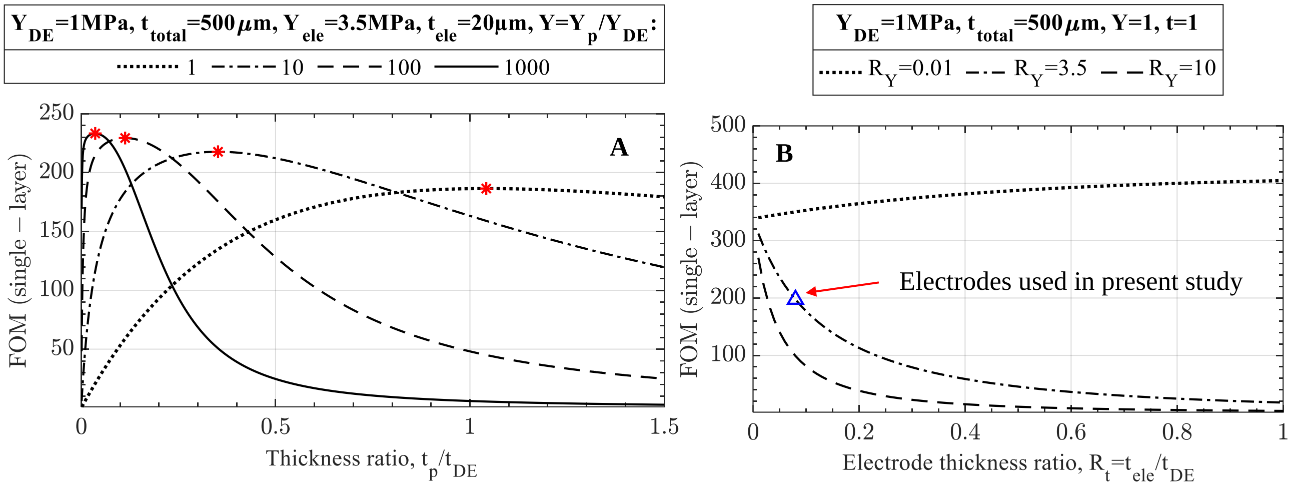

Supplemental Figure 4. Effects of electrodes on unimorph DEA actuation evaluated through the single-layer FOM for unimorph DEAs with (A) fixed total actuator thickness and electrodes, (B) fixed total actuator thickness and variable electrodes. For the fixed electrode, thickness and modulus are taken as for PEDOT:PSS-based electrodes used in present study.

3.4 Optimization of UDEA Designs Reported in Literature

Supplemental Figure 5 demonstrates application the derived FOM (for single-layer UDEAs) and analytical model (for multilayer UDEAs) for the reported UDEA designs in literature. The optimization represents adjusting the passive layer thickness for the reported materials of all the layers and thicknesses of DE layers and electrode. The curvatures (FOMs) of the reported actuators and their optimized design are used in Table 1 of the main manuscript.

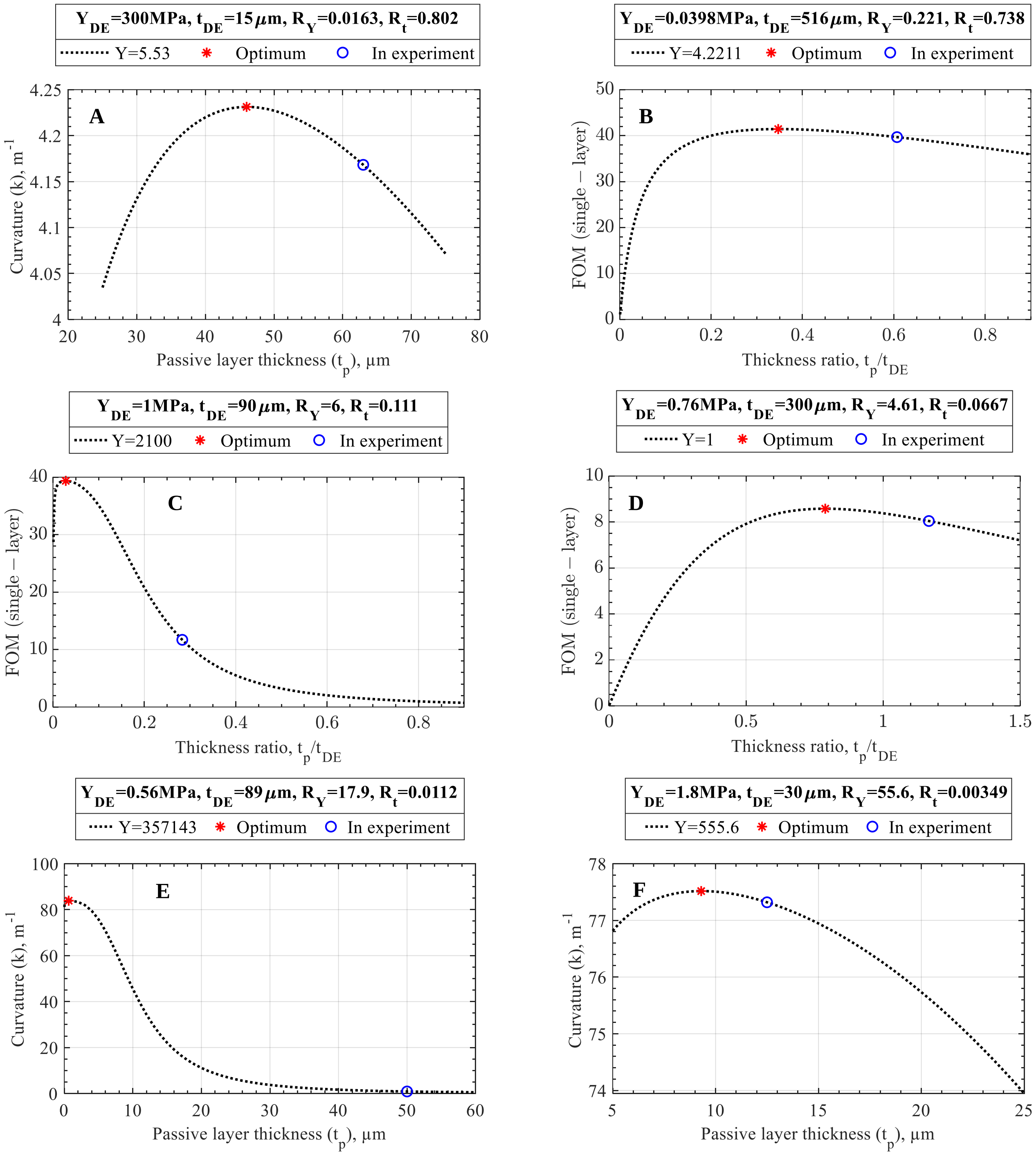

Supplemental Figure 5. Actuation performance of single-layer and multilayer UDEA designs in the literature with various thickness of their passive layers: (A) [6], (B) [8], (C) [15], (D) [5], (E) [11], (F) [16]. Actual reported designs and optimized designs are highlighted and used in Table 1 of the main paper.

4 Viscosity Characterization

4.1 Viscometer Testing

Viscosity values from the viscometer test of the silicone compositions were interpolated into viscosity-time functions. While functions of time only were obtained for Sylgard 182 and 184 due to their near-Newtonian behavior, additional step was performed to obtain correct functions for Sylgard 186 that account for shear strain dependence. Firstly, power-law fluid model coefficients (K, n) were determined by applying the below power-law fluid equation to the apparent viscosities measured at 0.27 and 0.027 1/s strain rate (3 and 0.3 rpm viscometer rotor speed, respectively). Strain rates were estimated as shown in **Error! Reference source not found.** and **Error! Reference source not found.**.

$$\mu_{app} = K\dot{\gamma}^{n-1}$$

Next, the coefficients were determined for each time step and interpolated by polynomials of various orders to select appropriate fit function (Supplementary Figure 6). Finally, quadratic polynomial was selected for coefficient n and cubic polynomial was selected for coefficient K. Same process was repeated for Sylgard 186 (40:1) to determine its viscosity-time function (Supplementary Figure 7). As can be seen, it is harder to accurately fit Sylgard 186 (40:1) coefficients, therefore, the simplest linear fit is selected for both coefficients.

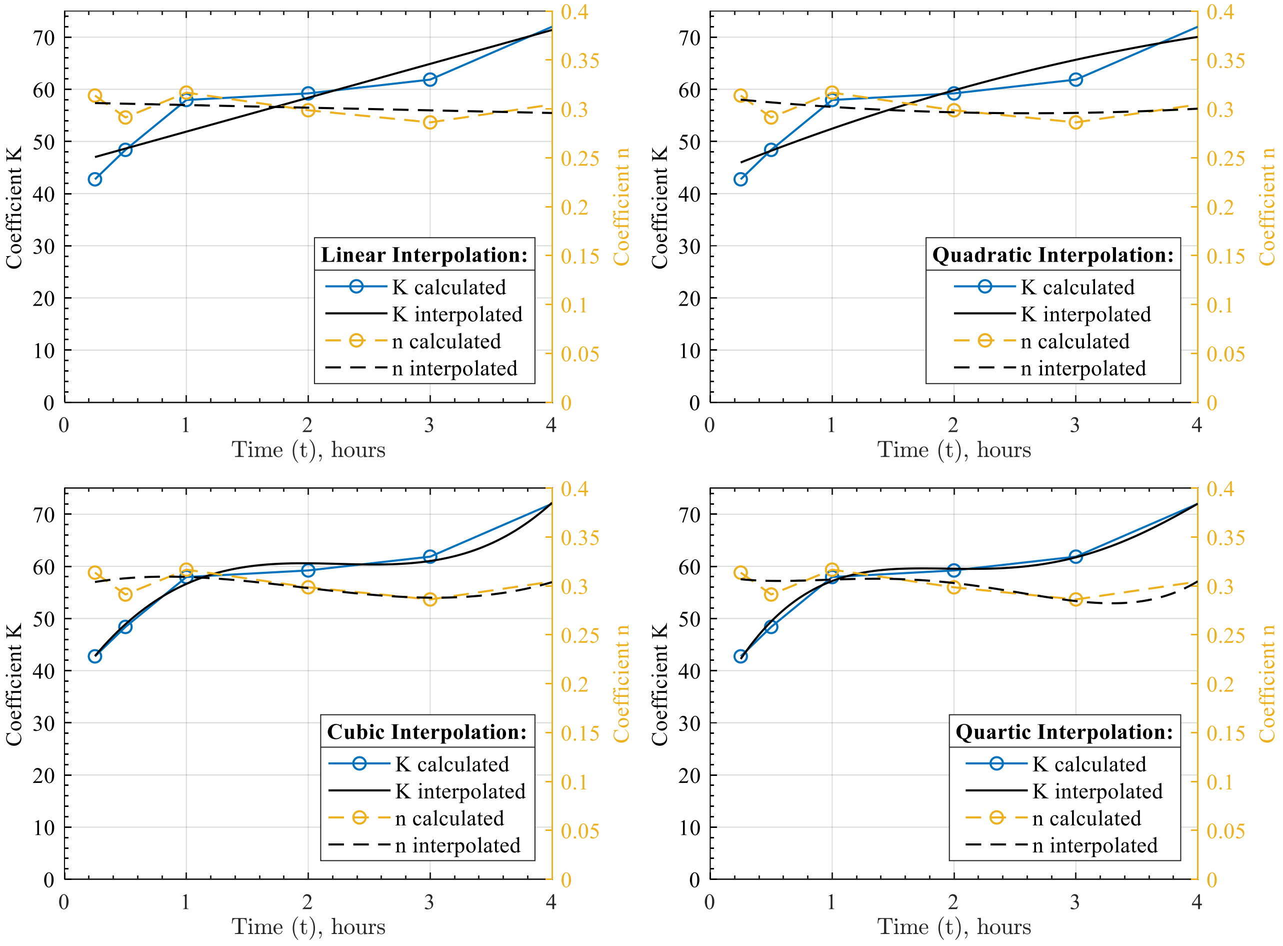

Supplementary Figure 6. Interpolating calculated values of K & n power-law fluid coefficients of Sylgard 186 (10:1) by different order polynomials.

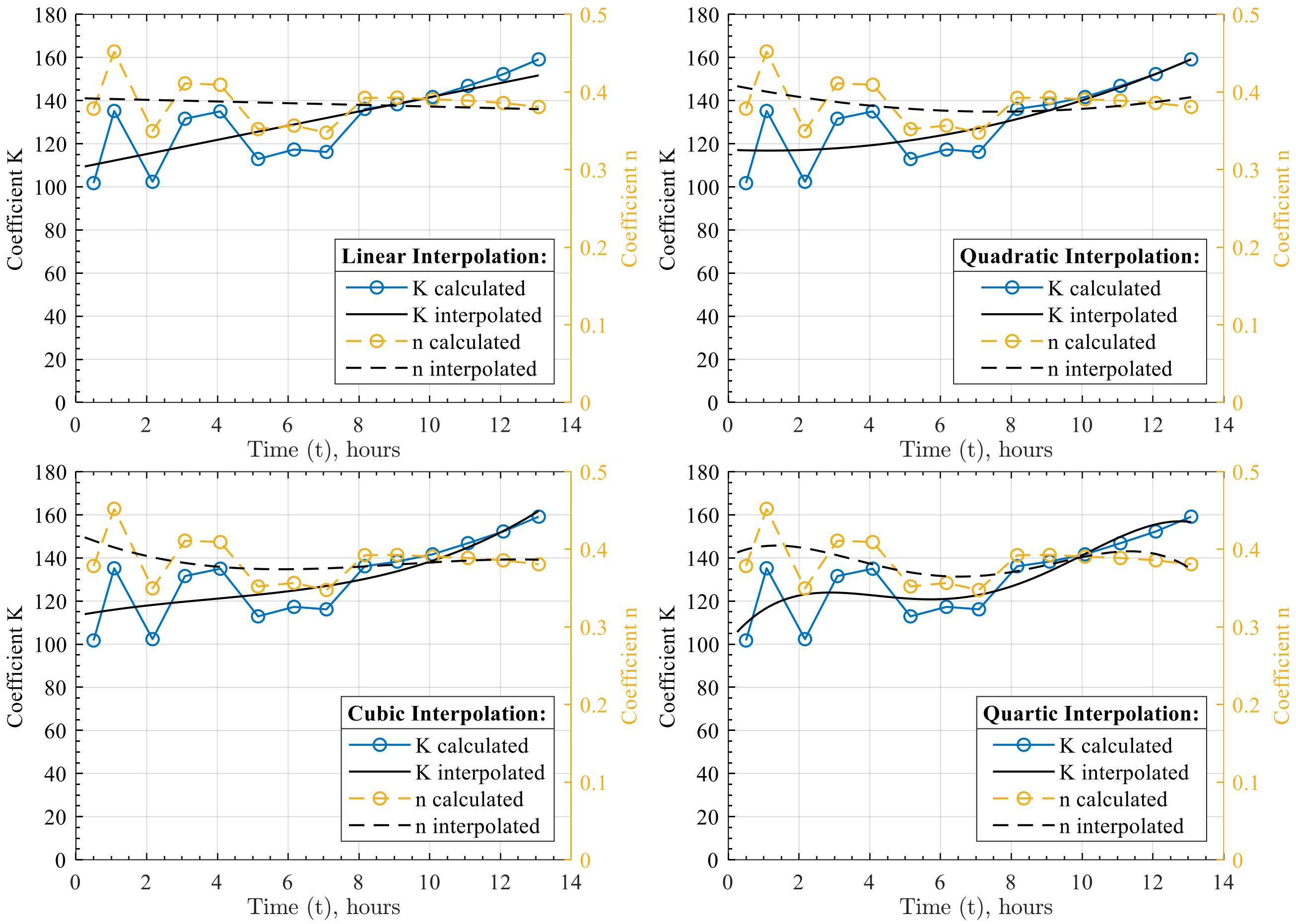

Supplementary Figure 7. Interpolating calculated values of K & n power-law fluid coefficients of Sylgard 186 (40:1) by different order polynomials.

Strain rates were estimated for both rotary viscometer test and contact dispensing of Sylgard silicones (**Error! Reference source not found.**). In both cases, silicones were treated as incompressible Newtonian fluids. While providing an approximate solution for velocity and strain rate distribution, close maximum values can be estimated for an uncomplicated comparison (**Error! Reference source not found.**).

Supplementary Table 1. Estimation of velocity distribution and strain rate in the viscometer and printing tests of Sylgard silicones as for incompressible Newtonian fluids.

Viscometer test	Printing test
$$v = Ar + \frac{B}{r}$$ where $A = \omega_1 \frac{\mu - \eta^2}{1 - \eta^2}, B = \omega_1 R_1^2 \frac{1 - \mu}{1 - \eta^2}$ where $\mu = \omega_2/\omega_1, \eta = R_1/R_2$ where R_1, ω_1 are radius and angular velocity of the viscometer rotor, R_2 is the cup radius, $\omega_2 = 0$ is the cup angular velocity, v is the tangential (azimuthal) velocity of the silicone at radius r.	$$v = 2\bar{v}\left(1 - \frac{r^2}{R^2}\right)$$ where $\bar{v}$ is the average velocity of the flow in a pipe (dispensing needle), R is the inner radius of the dispensing needle, v is the flow velocity at radius r.
**Velocity-strain rate relation for both cases**	

$\dot{\gamma} = \frac{1}{2}\left((\nabla v) + (\nabla v)^T\right)$ simplifies to $\dot{\gamma} = dv/dr$ for both cases, where $\dot{\gamma}$ is strain rate, ∇v is flow velocity gradient

After the first viscosity test with rotational viscometer, the second printing test determined the handling time of the mixed silicones on practice (Table 3). Supplementary Figure 9 shows the line patterns printed with various mixed silicones for up to 12 hours after material being mixed. The handling times were determined by judging the quality of the lines in terms on continuity and material dripping. While some lines are not printed in full because of imperfect parameters settings for the particular mixture, the real defects and inability to print starts when material starts clogging the dispensing needle and dripping. Additionally, effect of temperature of the printing bed on handling time of Sylgard 182(10:1) were studied (Supplementary Figure 10).

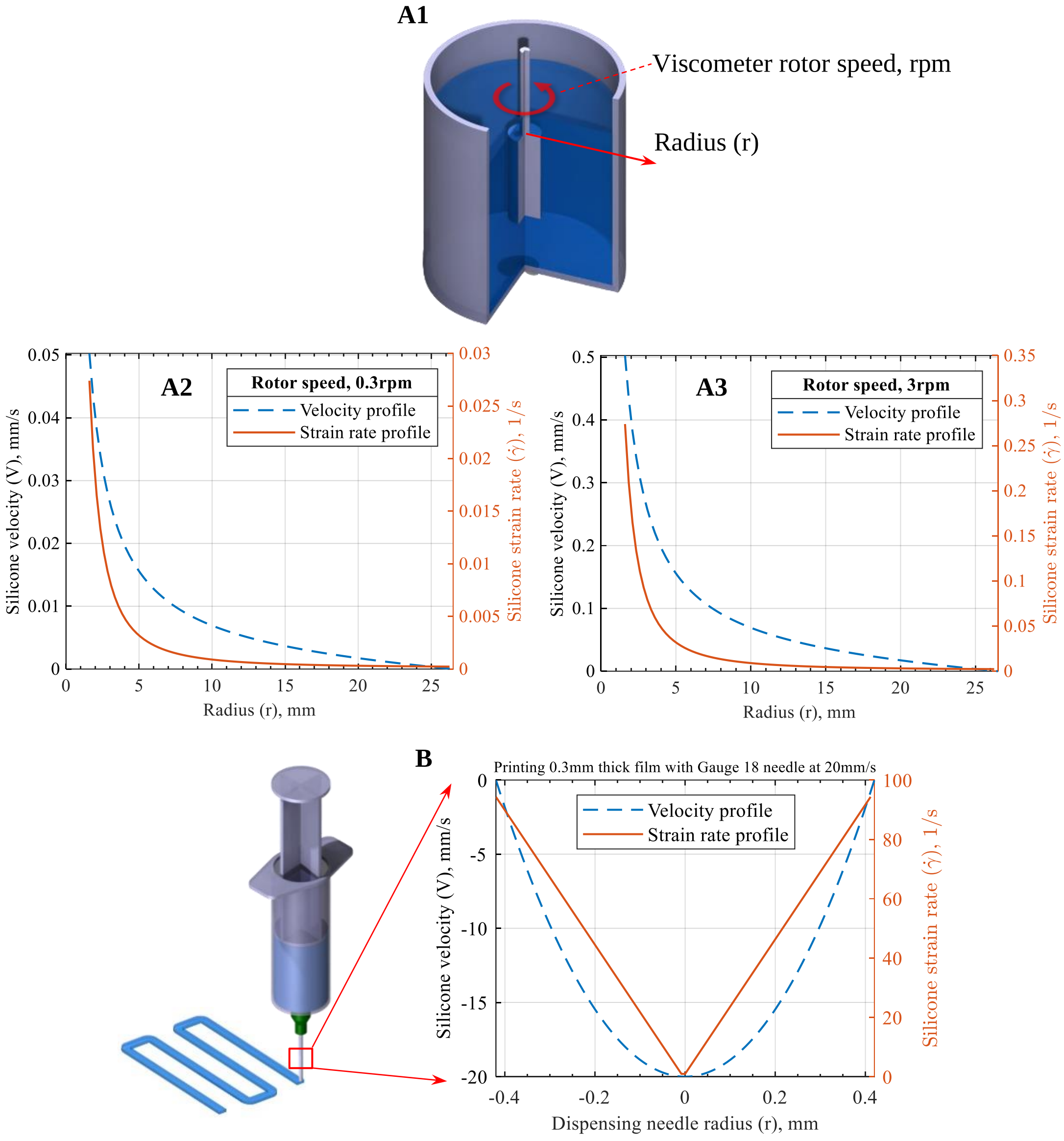

Supplementary Figure 8. Strain rates and velocity profiles of silicones in (A1-A3) viscometer test at various rotor speeds and (B) in the dispensing needle during 3D printing.

4.2 3D Printing Testing

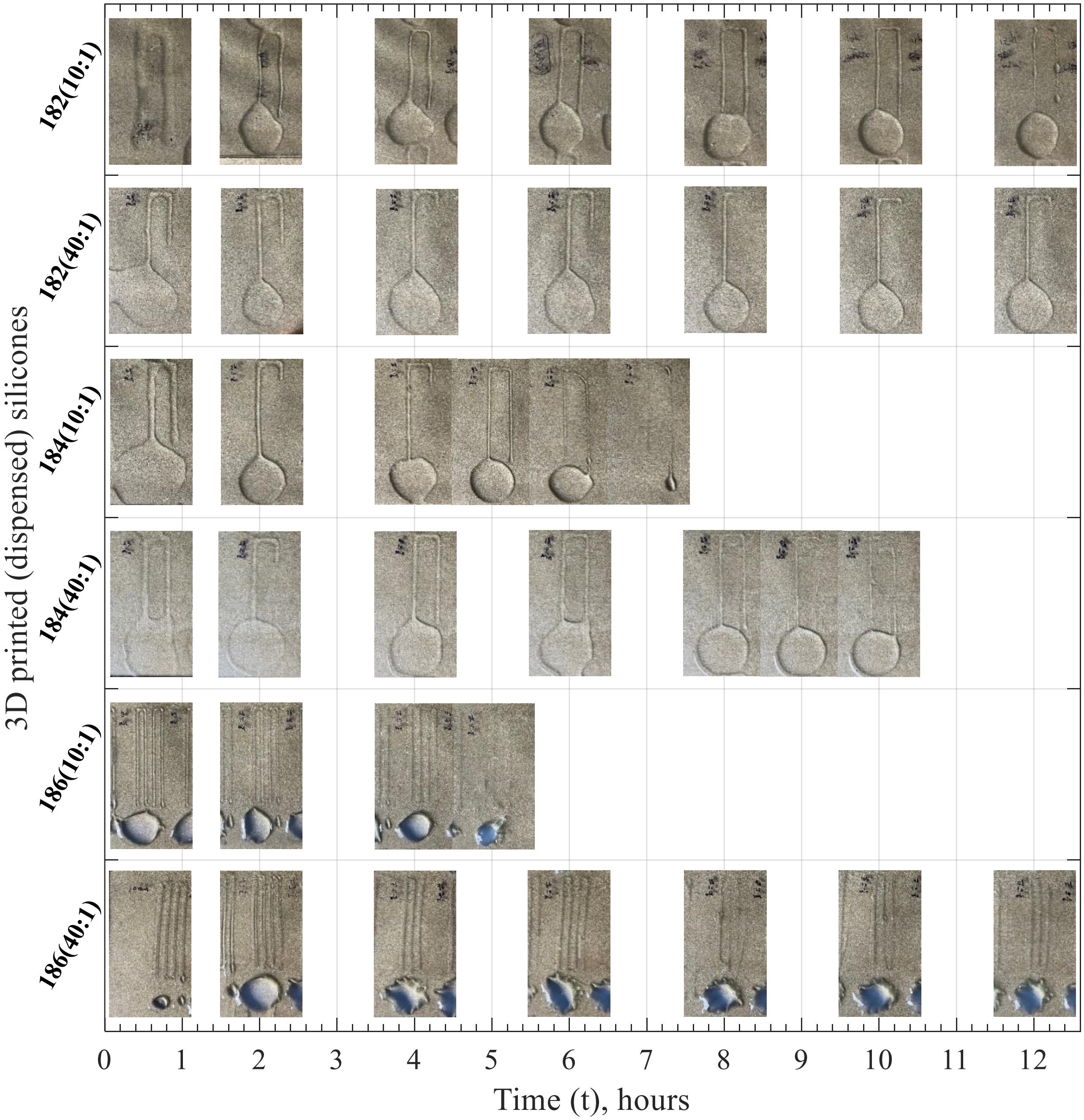

Supplementary Figure 9. 3D Printing (contact dispensing) test result evaluating rheology of the silicones Sylgard 182,184, and 186 mixed in the standard 10:1 ratio and non-standard 40:1 ratio.

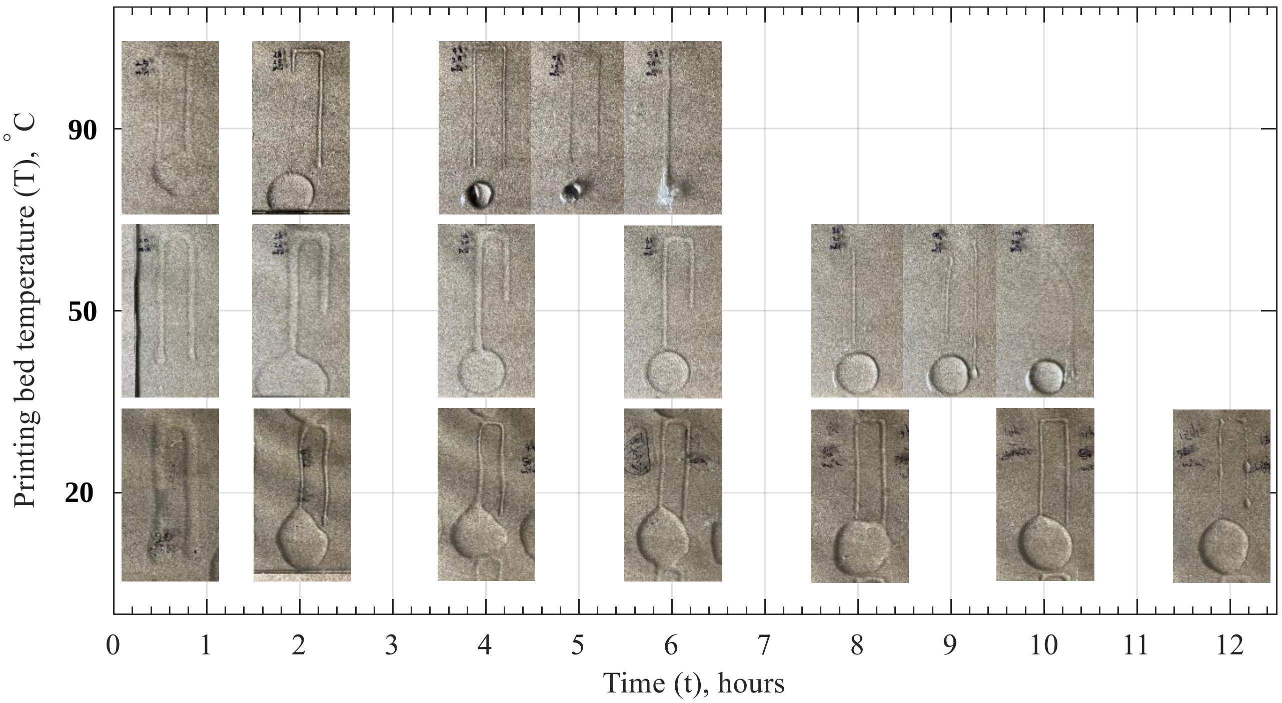

Supplementary Figure 10. 3D Printing (contact dispensing) test result evaluating rheology of the silicone Sylgard 182 mixed and printed at different printing bed temperatures.

5 Electromechanical Material Characterization

Tensile stress-strain curves are shown in Supplementary Figure 11a. The overall softening effect at various strains is achieved thanks to lower degree of polymerization (cross-linking of PDMS monomers). Furthermore, the stress-strain curves of weakly cross-linked PDMS, e.g., Sylgard 182 (30:1) and (40:1), are more linear compared to the S-shaped stress-strain curves of the highly cross-linked PDMS. As the initially stiffer region of the S-shaped stress-strain curve is hindered, enhanced softness at low strain can benefit applications that do not operate at high strains or do not utilize prestretch, e.g., most of 3D printed DEAs. Supplementary Figure 11b shows the determined values of tensile Young's modulus.

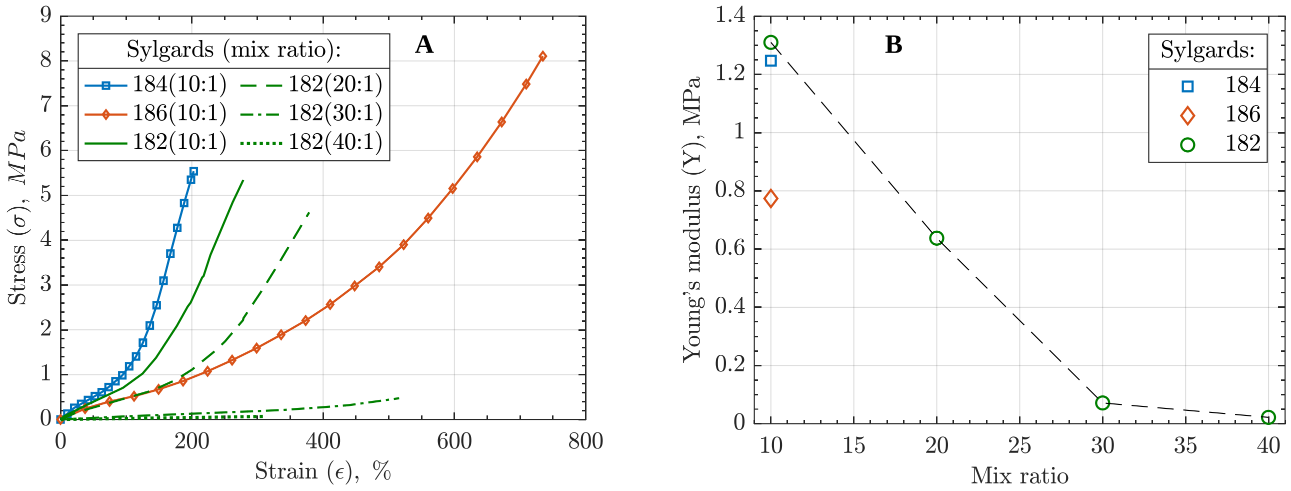

Supplementary Figure 11. (A) Tensile stress-strain curves and (B) Young's moduli of variously mixed silicones.

Table 2. Nanoindenter mechanical characterization results of the prepared silicone compositions.

	Sylgard 184 (10:1)	Sylgard 186 (10:1)	Sylgard 182 10:1	20:1	30:1	40:1
Reduced modulus, MPa	4.12	2.94	3.58	1.52	-	-
Young's modulus, MPa (assuming ν=0.49)	3.13	2.24	2.72	1.16	-	-
Hardness (measured), kPa	715.7	515.7	622.8	243.5	-	-
Hardness Shore A*	48.3	42.1	45.7	30.0	-	-
Hardness Shore A (manufacturer)	43	24	51	-	-	-

* Converted from the Young's modulus using the Neo-Hookean relation: $log_{10}E = 0.0235 \cdot S - 0.6403$, where E is Young's modulus in MPa and S is the ASTM D2240 type A hardness.

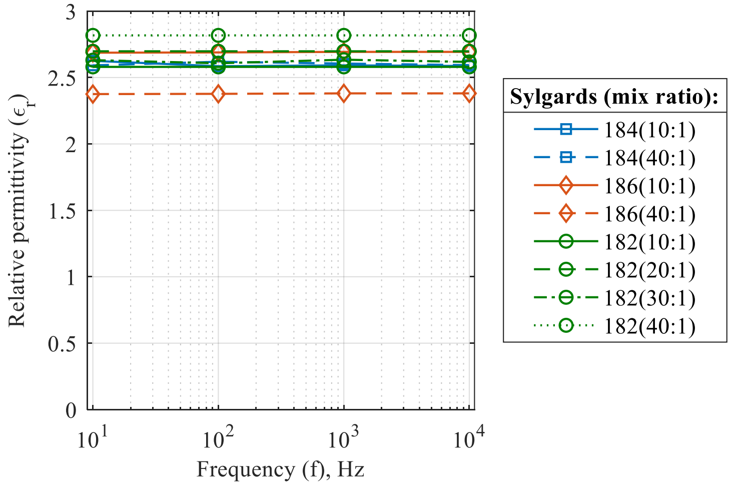

Supplementary Figure 12. Permittivity independence on frequency within the range of 1 Hz to 10 kHz of the prepared silicones.

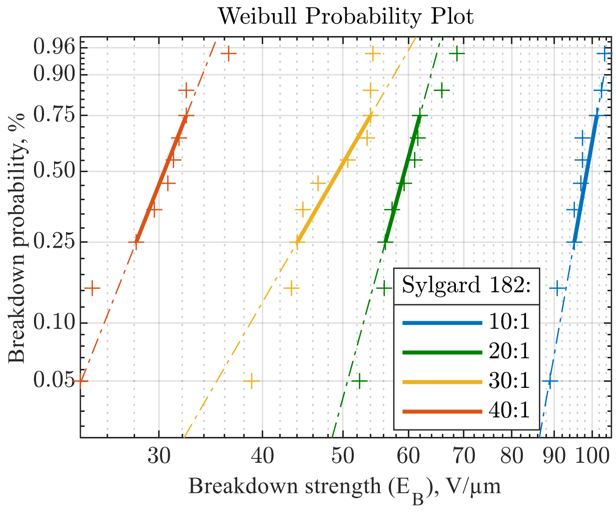

Supplementary Figure 13. Weibull Probability Plot for breakdown strength of various mix ratios of Sylgard 182.
